# Transcriptome-Wide Survey of Mouse CNS-Derived Cells Reveals Monoallelic Expression within Novel Gene Families

**DOI:** 10.1371/journal.pone.0031751

**Published:** 2012-02-22

**Authors:** Sierra M. Li, Zuzana Valo, Jinhui Wang, Hanlin Gao, Chauncey W. Bowers, Judith Singer-Sam

**Affiliations:** 1 Division of Biostatistics, Beckman Research Institute, City of Hope National Medical Center, Duarte, California, United States of America; 2 Division of Biology, Beckman Research Institute, City of Hope National Medical Center, Duarte, California, United States of America; 3 Division of Computational Biology, Beckman Research Institute, City of Hope National Medical Center, Duarte, California, United States of America; Ohio State University Medical Center, United States of America

## Abstract

Monoallelic expression is an integral component of regulation of a number of essential genes and gene families. To probe for allele-specific expression in cells of CNS origin, we used next-generation sequencing (RNA-seq) to analyze four clonal neural stem cell (NSC) lines derived from *Mus musculus* C57BL/6 (B6)×*Mus musculus molossinus* (JF1) adult female mice. We established a JF1 cSNP library, then ascertained transcriptome-wide expression from B6 *vs.* JF1 alleles in the NSC lines. Validating the assay, we found that 262 of 268 X-linked genes evaluable in at least one cell line showed monoallelic expression (at least 85% expression of the predominant allele, *p*-value<0.05). For autosomal genes 170 of 7,198 genes (2.4% of the total) showed monoallelic expression in at least 2 evaluable cell lines. The group included eight known imprinted genes with the expected pattern of allele-specific expression. Among the other autosomal genes with monoallelic expression were five members of the glutathione transferase gene superfamily, which processes xenobiotic compounds as well as carcinogens and cancer therapeutic agents. Monoallelic expression within this superfamily thus may play a functional role in the response to diverse and potentially lethal exogenous factors, as is the case for the immunoglobulin and olfactory receptor superfamilies. Other genes and gene families showing monoallelic expression include the annexin gene family and the *Thy1* gene, both linked to inflammation and cancer, as well as genes linked to alcohol dependence (*Gabrg1*) and epilepsy *(Kcnma1)*. The annotated set of genes will provide a resource for investigation of mechanisms underlying certain cases of these and other major disorders.

## Introduction

Monoallelic expression (also termed allelic exclusion) refers to the epigenetic silencing of one of the two copies (i.e., alleles) of a gene. Well-studied examples of monoallelic expression in placental mammals include X-inactivation, which affects most genes on an entire chromosome, and imprinting, the parent-of-origin silencing of some autosomal genes. In the case of X-inactivation in females, there is random silencing of one X chromosome in individual cells early in development, and the pattern of silencing is stably inherited through all subsequent somatic cell divisions.

Other genes known to show monoallleic expression can be broadly classified into two categories. The first group includes immunoglobulin and olfactory receptor genes [1], [2]. Genes in this group are expressed at high level from a single allele following induction/differentiation. While the mechanisms leading to allele-specific expression differ between immunoglobulin and olfactory receptor gene families, they share intriguing similarities. Monoallelic expression in both cases increases the specificity of a given responder cell as well as the potential diversity within a mixed population of such cells. The two superfamilies also share a similar chromosomal distribution, with multi-gene clusters distributed on multiple chromosomes. Notably, both superfamilies form part of an individual's defense against highly variable, potentially lethal external agents.

A second category of genes is expressed at low levels prior to induction (e.g. IL-4 or IL-5 in latent T cells) [3]. Following stimulation, the expression level may be greatly increased, accompanied by a shift from monoallelic to biallelic expression. In such cases, allele-specific silencing is said to be stochastic, i.e., variable, in cells of the same lineage [4].

Hybridization-based studies have shown that at least 1% of autosomal genes show a pattern of random monoallelic expression in human lymphoblastoid cells [5] and in mouse neural stem cells [6], [7]. In this study we used next-generation RNA sequencing (RNA-seq), which offers major advantages over previous methods for transcriptome-wide analysis of random allele-specific expression. In addition to its accuracy and sensitivity [8], RNA-seq allows expression profiling and cSNP analysis to be accomplished in the same experiment, so that allele-specific expression can be correlated with mRNA abundance (and potential biological significance). Using the Illumina sequencing platform for RNA-seq analysis of mouse tissues, Mortazavi et al. used “reads per kilobase of exon model per million mapped reads” (RPKM) to quantify transcript levels, and showed that the linear range of transcript detection extended over five orders of magnitude (0.1∼10,000 RPKM) [9]. More recent studies using the same platform include an analysis of escape from X-inactivation [10] and surveys of imprinting in mouse brain [11]–[13].

In this study, we analyzed RNA-seq data using multiple clonal cell lines, and identified monoallelic expression with a low false discovery rate (FDR). Use of these clonal neural stem cell (NSC) lines derived from F_1_ mice of both reciprocal crosses allowed us to distinguish imprinting from random allelic exclusion. It also allowed us to detect monoallelic expression that might be missed in a single cell line, since, as discussed above, allelic exclusion is frequently stochastic, i.e., variable in different founder cells (5–7). Our results confirm allele-specific expression of ∼2.5% of autosomal genes in CNS-derived stem cells [7]. Furthermore, the annotated list includes genes whose monoallelic expression may be relevant to a number of major inherited disorders of the CNS, as well as disorders that can arise from somatic mutations, including various cancers.

## Results and Discussion

### Transcriptome-wide Profiling of Allele-specific Expression by cSNP-seq

**Figure 1:**
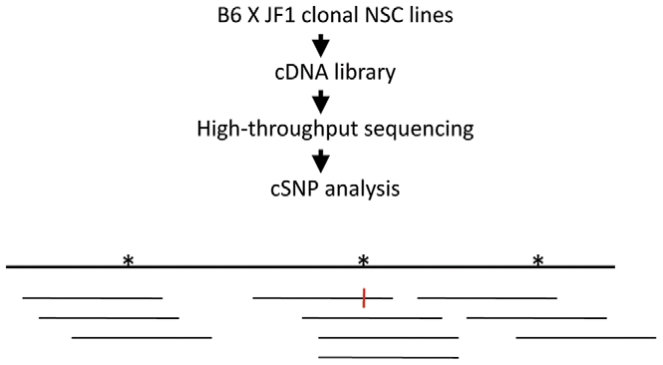
Outline of the cSNP-seq assay. The diagram shows a hypothetical single-exon gene with 3 SNPS (asterisks), a pooled SNP depth of 10, and a 90% preference for the B6 allele (the red vertical bar shows a JF1 sequence at the cSNP indicated.).

To perform a transcriptome-wide search for random monoallelic expression in cells originating in the CNS, we made use of four clonal NSC lines isolated as previously described [7]. The cells are derived from adult forebrain of female mice resulting from the cross Mus musculus C56BL/6 (B6)×Mus musculus molossinus JF1 (JF1), and retain the potential to differentiate to populations of neurons and astrocytes [6]. Of the NSC lines analyzed, 2A1, 3A1 and 4A5a were derived from B6^MAT^×JF1^PAT^ hybrid mice; the cell line 2A5 was derived from the reciprocal cross, JF1^MAT^×B6^PAT^. We used next-generation sequencing, first to create a cSNP library for JF1 brain, then to analyze allele-specific expression in each of the four NSC lines (“cSNP-seq analysis”). In each case, polyA^+^ RNA was converted to double-stranded cDNA. Following addition of linkers specified by Illumina, 250–300 bp fragments were analyzed. For JF1, we analyzed ∼132 million single or paired-end reads of length 40–80 bases. Of these, approximately 25–30% high quality reads without gaps could be uniquely aligned to the Refseq transcriptome. We used a threshold of RPKM> = 3 to distinguish expressed genes from background [9]. Of 22,088 Refseq genes expressed in JF1 brain or hybrid cell lines, ∼10,000 genes in the JF1 sample had an RPKM> = 3, while ∼8,500 genes met the same threshold in each NSC line. Of these, we considered genes to be statistically evaluable if the pooled SNP depth of coverage was at least 10 (Figure 1). Approximately 7100 genes in NSC cells (range: 6925–7373 genes) met these two thresholds, i.e., RPKM> = 3 and pooled SNP depth of coverage > = 10. For these genes, we computed relative B6 and JF1 expression (*P_B6_* and *P_JF1_*, respectively). Genes were considered to show monoallelic expression if *P_B6_* or *P_JF1_* was > = 0.85, with a *p*-value< = 0.05 (exact binomial test, null hypothesis: *P_B6_* = *P_JF1_* = 0.5). With the same *p*-value threshold, another subset of genes for which 0.7< = *P_B6_* or *P_JF_1*<0.85 were considered to show a trend towards monoallelic expression. Genes for which *P_B6_* or *P_JF_1*<0.7 were considered to show biallelic expression. A small set of genes (∼40–90 in each cell line) were excluded from classification because although *P_B6_* or *P_JF_1*> = 0.7, the results were not statistically significant (*p*-value>0.05), mostly due the low depth of SNP coverage of the genes. Details of the statistical analysis are in Methods S1. Results for all evaluable genes as well as summaries of Illumina sequencing and mapping are shown in Table S1, Figure S1 and Dataset S1.

### Proof-of-principle: X-linked Genes

**Figure 2:**
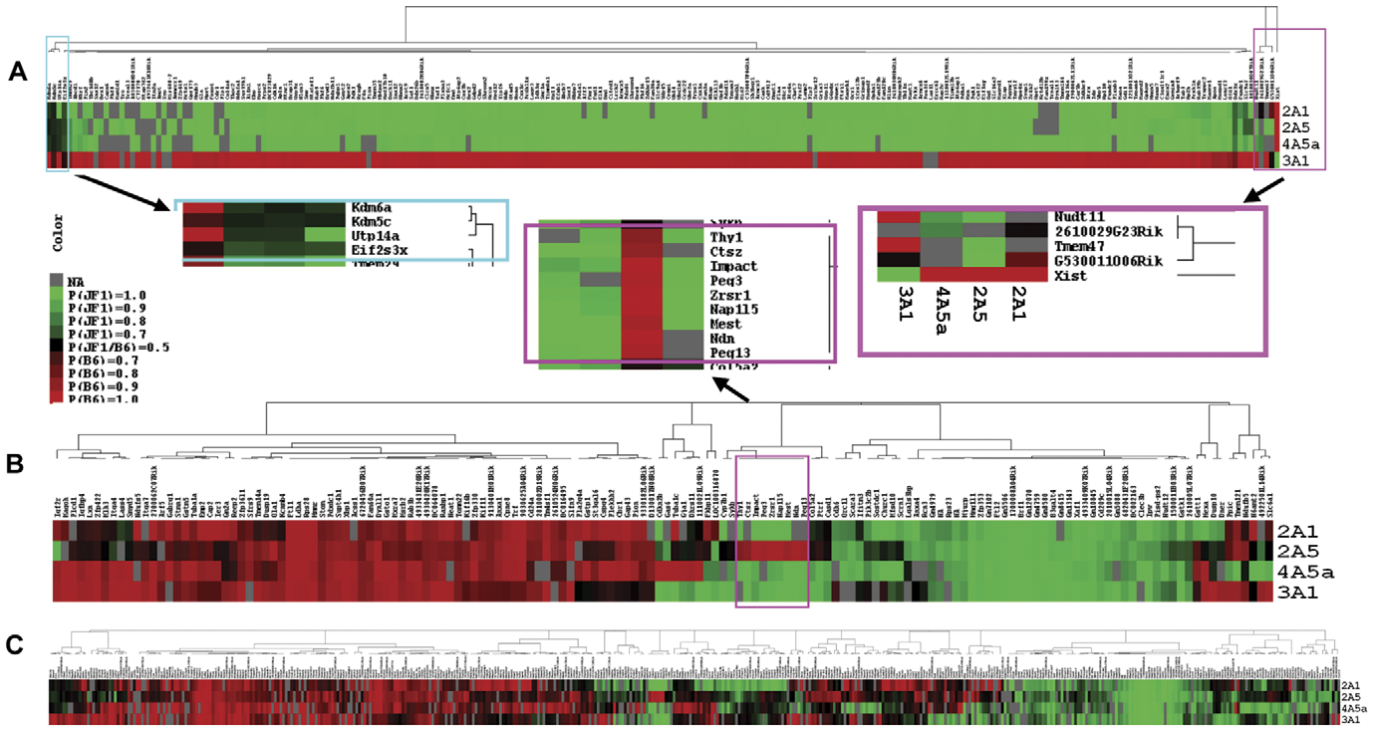
Heatmaps showing results of cSNP analysis. A. X linked genes (n = 235) evaluable for at least 2 out of the 4 cell lines. Insets expand results of hierarchical clustering. *Right inset*: Unique pattern of the *Xist* gene. *Left inset:* Genes that escape X-inactivation (see text). B. Autosomal genes (n = 152) that show monoallelic expression in at least 2 of 3 evaluable cell lines. *Inset:* Genes that show imprinted gene expression. C. Autosomal genes (n = 476) with monoallelic expression in at least 1 of 2 evaluable cell lines.

To establish the validity of the method, we first analyzed X-linked genes in the NSC lines. A total of 215, 222, 243 and 211 Refseq genes were evaluable in cell lines 2A1, 2A5, 3A1 and 4A5a, using the criteria described above. Taken together, 268 genes were evaluable in at least one cell line, of which 262 genes showed monoallelic expression (97.8%), and 235 genes were evaluable in at least 2 cell lines, of which 231 and 229 showed monoallelic expression in at least one (98.3%) or two (97.4%) lines. The estimated FDR ranged from 0.24% (cell line 2A5) to 2.25% (cell line 4A5a) as shown in Table S1. Figure 2A shows the heatmaps for the expression pattern for the 235 genes evaluable in at least 2 cell lines, with clustering based on strength of monoallelic expression. Each cell line shows predominant expression from only one X chromosome: the JF1-derived X chromosome for cell lines 2A1, 2A5 and 4A5a, and the B6-derived X chromosome for cell line 3A1. *Xist* is in a subcluster by itself since, as expected, it shows the reverse pattern of the other X-linked genes in each cell line. Several genes, clustered at the left corner of the heatmap, show biallelic or less strong monoallelic expression patterns. The cluster contains 3 previously known escape genes: *Kdm6a, Kdm5c* and *Eif2s3X*, and also includes *Utp14a*, a small nucleolar p53-binding protein involved in 18 s rRNA maturation [14]. This gene, which shows biallelic expression in two of the four cell lines, maps to XqA4, not in the vicinity of the other known escape genes.

### Allele-specific Expression of Autosomal Genes

On autosomes, ∼7000 genes were evaluable in each cell line (range: 6724–7151), with ∼200 genes (range: 172–244) showing monoallelic expression. The FDR varied from 1.33% to 3.75% among the 4 hybrid cell lines. Of the 7,198 autosomal genes evaluable in at least 2 cell lines, 476 showed monoallelic expression in at least 1 cell line, of which 170 genes (2.4%) showed monoallelic expression in at least 2 cell lines. Among the 170 genes, 152 genes were evaluable in at least 3 cell lines. The heatmap shown in Figure 2B summarizes results for these 152 genes. Among the genes was a cluster that includes the known paternally expressed genes, *Impact, Peg3, Zrsr1, Nap1l5, Mest, Ndn* and *Peg13* (Figure 2B inset). We found that the two novel potentially imprinted genes in the cluster, *Ctsz* and *Thy1*, are not imprinted in mouse brain. The known maternally expressed gene, *Igf2r* is in a unique subcluster at the extreme left of the heatmap.

Of the remaining ∼140 genes, most show a strain- or sequence-specific pattern, i.e., the same allele (B6 or JF1) is favored in each cell line that shows monoallelic expression, similar to a pattern noted previously [15]. An additional group of ∼20 genes shows a random pattern, i.e., the gene is expressed from the B6 allele, the JF1 allele or both alleles, depending upon the cell line. The variable pattern of monoallelic and biallelic expression across several cell lines is consistent with previous studies of autosomal monoallelic expression [4]–[6]. The heatmap in Figure 2C shows a similar pattern for 476 genes with monoallelic expression in at least 1 of 2 evaluable cell lines.

Previous studies have shown that for specific genes expressed from either one or both alleles, the expression level is frequently higher for cells showing biallelic expression [5], [6]. Our study allowed us to examine transcriptome-wide differences in expression levels associated with monoallelic vs. biallelic expression. We found, by two statistical analyses, that monoallelic expression is correlated with a ∼30–35% reduction in expression level across a broad range of RPKM.

**Figure 3:**
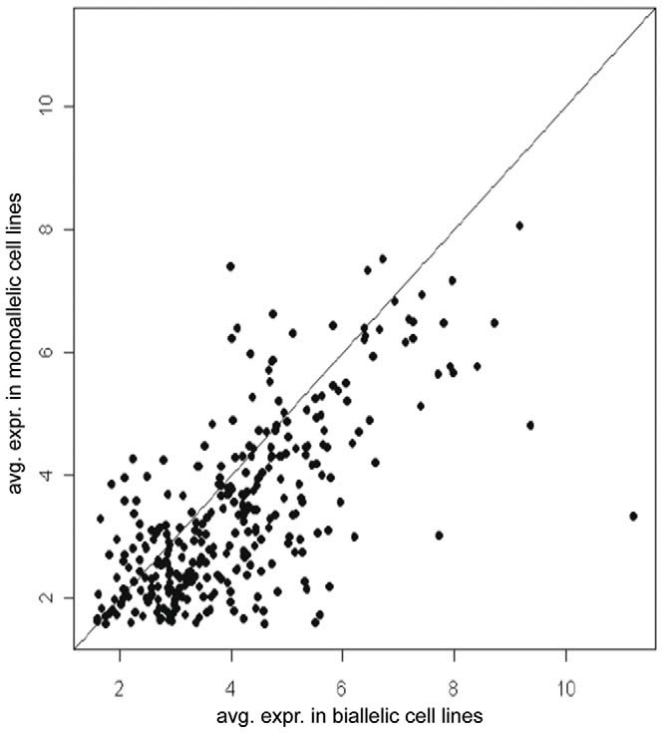
Expression levels *vs.* monoallelic or biallelic expression. Results are plotted for 313 genes that show monoallelic expression in some NSC lines and biallelic expression in others. For each gene the average log2(RPKM) in NSC lines with monoallelic expression (y-axis) is plotted vs. the log2(RPKM) in cell lines with biallelic expression.

In the first analysis, for the 313 genes that showed a mixed pattern of biallelic expression in some cell lines and monoallelic expression in others, we compared average expression levels in the respective cell lines. Figure 3 shows that there is a trend towards lower RNA abundance in cell lines expressing only one allele. The mean transcript level of a gene in cells with monoallelic expression was 64.17% that of cells with biallelic expression (i.e., the mean log2(RPKM) difference was 0.64 (95% CI 0.51–0.78), which is statistically significant by the paired t-test at a *p*-value of 2.2e^−16^). If the number of expressed alleles were the only factor, the predicted mean transcript ratio would be 0.5. Therefore, our finding of a ratio of 0.64 suggests modulation by additional regulatory elements. These results are comparable to the effect of gene dosage on global gene expression [16], [17].

We found a similar statistically significant ∼30% difference when we compared expression levels (RPKM) for genes that showed monoallelic vs. those that showed biallelic expression in each cell line (Figure S2). In both statistical analyses, the broad distribution of RPKM for genes showing monoallelic as well as biallelic expression demonstrates that a sizable number of genes with allelic exclusion are expressed at moderate to high levels.

To determine whether the genes showing monoallelic expression were clustered on particular autosomes, we plotted the chromosomal distribution of these genes in each NSC line (Figure S3). The genes showing single-allele expression are broadly distributed on all autosomes, rather than confined to specific chromosomes or chromosomal regions. These results are consistent with an absence of chromosome-wide coordination for autosomal monoallelic expression [5].

### Enriched Monoallelic Expression of Specific Gene Families: Glutathione Transferases, Annexins and Protocadherins

We used the DAVID Bioinformatics Resource to search for enrichment of particular categories of genes. Considering protein domains and Kegg pathways, we found several categories of genes to be enriched among the genes showing monoallelic expression in at least 2 cell lines (Table S2). Included were five genes of the glutathione superfamily (∼17-fold enrichment, FDR 1.21% to 2.37%). When genes showing monoallelic expression in at least 1 cell line were analyzed (n = 577), additional genes appeared, including 4 genes of the annexin gene superfamily (∼12-fold enrichment, FDR ∼6%). Notably, protocadherins also appeared (7–15 fold enrichment, FDR<10^−6^%). These members of the cadherin superfamily provide additional positive controls, since they are known to show monoallelic expression [18]. A recent study suggests that monoallic expression of protocadherins may contribute to specificity of neuronal circuitry [19].

### Monoallelic Expression of Gst superfamily Genes Following Differentiation of NSC Lines

Proteins of the abundant glutathione transferase (Gst) superfamily catalyze the addition of the tri-peptide glutathione to electrophilic compounds, resulting in detoxification of chemical carcinogens and pollutants, and also a decrease in the efficacy of some anticancer therapeutics (reviewed in Hayes et al. [20]). Gst proteins have a major additional role in adaptation to oxidative and inflammatory stress, resulting from the same catalytic activity applied to endogenous intermediates of the stress response.

Gst proteins are found in the cytosol (including the Gsta, Gstm, Gstp, Gstt gene families), mitochondria (the Gstk family) and microsomes. The different families, which are categorized based on sequence similarity, map to different clusters on multiple chromosomes, but the genes within each family are clustered at the same location. Gst proteins are typically dimers, with proteins in the Gsta andGstm families capable of forming heterodimers with other subunits in the same group.

There have been many reports of altered Gst expression in tumors (see, for example, Hayes and Pulford [21]). Because of the broad, overlapping specificity of Gst genes, knockout experiments of a single subunit have not always resulted in dramatic defects. In an important experiment, homozygous knockout of the 2 subunits of the mouse Gstp complex was shown to result in increased skin tumorigenesis in response to carcinogens and tumor promoters [22].

**Figure 4:**
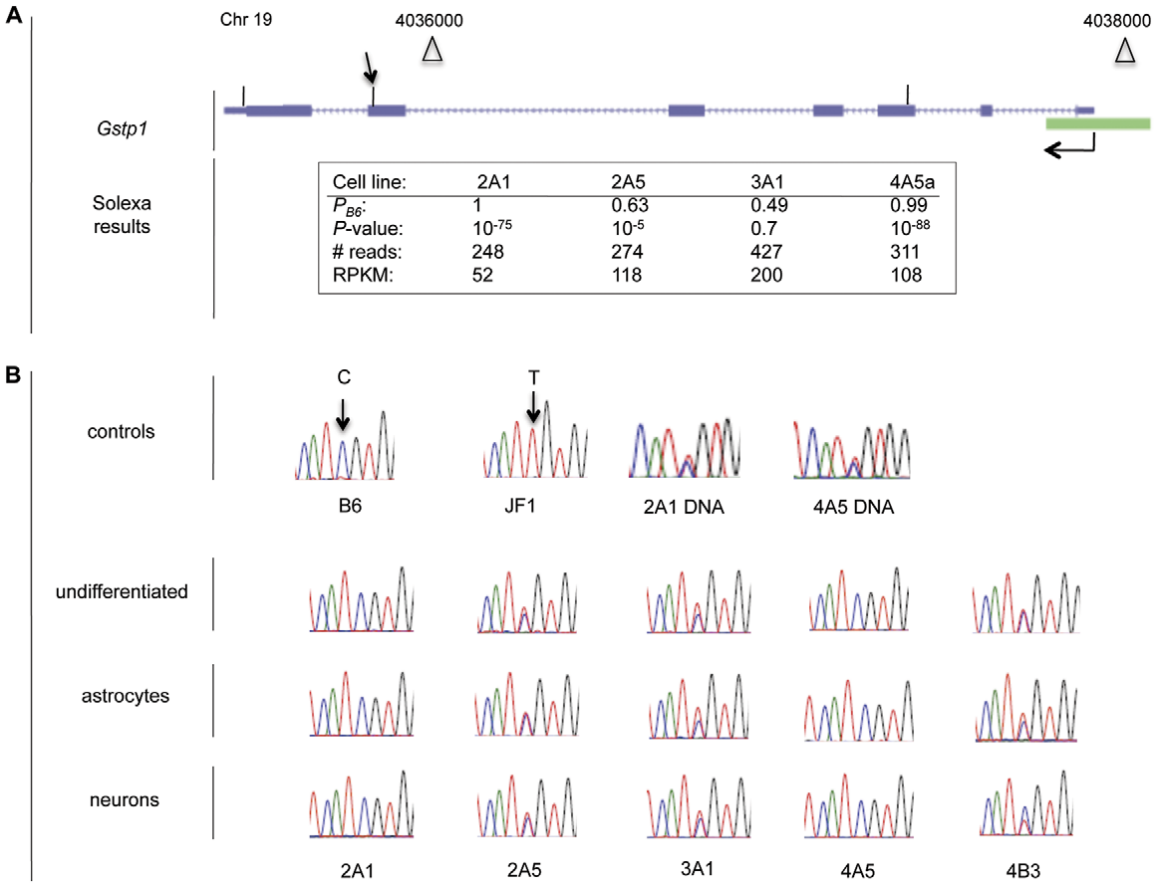
Allele-specific expression of *Gstp1*. A. Structure of the gene and results of Illumina sequencing in four NSC lines. The chromosomal location, start site and orientation of transcription, the 7 exons, and a CpG island (green rectangle) at the 5′ terminus are shown. The cSNPs in exons 3, 6 and 7 are indicated by the vertical lines above the respective exons. The table shows that the B6 allele is expressed in cell lines 2A1 and 4A5a, while both alleles are expressed in cell lines 2A5 and 3A1. B. RT-PCR results. *First row:* Chromatograms show a C/T cSNP between B6 and JF1 transcripts, and the presence of both alleles in genomic DNA of NSC lines 2A1 and 4A5. The arrow in Figure 4A indicates the location of the cSNP. *Second to fourth rows:* Analysis of RNA isolated from five NSC lines in the undifferentiated state, and differentiated to astrocytes and neurons, as indicated. The B6 allele is expressed in cell lines 2A1 and 4A5, while both alleles are expressed in cell lines. 2A5, 3A1 and 4B3.

Because of the compelling interest of the Gst superfamily, we used RT-PCR to analyze further five Gst genes that showed monoallelic expression by Illumina sequencing: *Gstk1, Gstm5, Gsto1, Gstp1* and *Gstt1*, each from a different subfamily. Table 1 confirms that the genes show monoallelic expression in multiple cell lines. Moreover, when the cell lines are differentiated to astrocytes and neurons, the allele-specific pattern of expression is almost entirely preserved. An example of the results obtained by the two independent methods is shown for the *Gstp1* gene in Figure 4. Figure 4A shows the location of the three cSNPs (in the 3^rd^, 6^th^ and 7^th^ exons, respectively) for which data was obtained by Illumina sequencing. The inset shows that two of the cell lines express the B6 allele only, while two others show biallelic expression. The RPKM indicate high levels of expression, approaching that of housekeeping genes in the four cell lines, even in the cell lines showing B6 expression only. On average, the RPKM are two-fold higher in the two lines showing biallelic expression, consistent with the results shown in Figure 3. Figure 4B shows the results of automated sequencing following RT-PCR with primers flanking one of the *Gstp1* cSNPs. Results are shown for NSC lines prior to and following differentiation to astrocytes and neurons. Complete RT-PCR results for all of the Gst genes assayed are shown in Figure S4.

**Table 1:** RT-PCR analysis of allele-specific expression of the Gst gene family in undifferentiated NSCs, and following differentiation to neurons and astrocytes.

Gene	Cell type	Neural stem cell line				
		2A1	2A5	3A1	4A5	4B3
*Gstk1*	*undiff*	100% J††	biallelic†	100% J††	100% J	100% J
	*astro* **	100% J	89% J	100% J	100% J	100% J
	*neurons* **	100% J	92% J	100% J	100% J	100% J
*Gstm5*	*undiff*	98% B††	biallelic†	86% B††	100% B	99% B
	*astro* **	100% B	80% B	biallelic	95% B	100% B
	*neurons* **	100% B	94% B	86% B	100% B	100% B
*Gsto1*	*undiff*	100% B††	84% B†	100% B††	100% B	100% B
	*astro* **	100% B	86% B	100% B	90% B	100% B
	*neurons* **	100% B	100% B	98% B	100% B	100% B
*Gstp1*	*undiff*	100% B††	biallelic†	biallelic†	100% B	biallelic
	*astro* **	100% B	biallelic	biallelic	100% B	biallelic
	*neurons* **	100% B	biallelic	biallelic	100% B	biallelic
*Gstt1*	*undiff*	100% J††	biallelic†	biallelic†	96% J	92% B
	*astro* **	97% J	91% B	*n.d.*	100% J	100% B
	*neurons* **	94% J	biallelic	biallelic	100% J	100% B

Numerical values are shown for all RT-PCR samples with at least a 0.8 preference for the B6 (B) or JF1 (J) allele. Other values are listed as biallelic (see Figure S4 for representative data and technical replicates.) *n.d.*, not detectable.

**Asterisks denote concordance with undifferentiated NSCs, *p*-value<0.001.

Daggers indicate results for samples evaluated by Illumina sequencing:

† Biallelic expression at *P_B6_ or P_JF1_*<0.85,

†† Monoallelic expression at *P_B6_* or *P_JF1_*> = 0.85.

### Additional Genes of Interest Showing Monoallelic Expression

Table 2 lists the five Gst genes as well as additional genes of interest validated by allele-specific RT-PCR. Results for these additional genes are shown in Figure S4 and summarized in Table S3.

**Table 2:** Selected list of autosomal genes with monoallelic expression by Illumina sequencing validated by RT-PCR.

Gene	Mono/Eval NSCs†	RPKM		Chr	Description
		Mono NSCs	JF1 brain		
*Anxa1*	3/4 (B6)	10 to 78	2	19	Annexin I, anti-inflammatory pathway
*Anxa2*	1/4 (B6)	77	63	9	Annexin II, cell growth, signal transduction
*Chl1*	1/4 (JF1)	37	48	6	Cell adhesion molecule (L1CAM homolog)
*Gabrg1*	2/3 (B6)	5 to 6	18	5	GABA A receptor, gamma 1 (alcoholism)
*Gm2a*	2/4 (B6)	39 to 66	55	11	GM2-gangloside activator
*Gstk1*	3/4 (JF1)	11 to 22	15	6	Glutathione S-transferase, kappa 1
*Gstm5*	3/4 (B6)	49 to 91	70	3	Glutathione S-transferase, mu 5
*Gsto1*	3/4 (B6)	17 to 20	30	19	Glutathione S-transferase, omega 1
*Gstp1*	2/4 (B6)	52 to 200	108	19	Glutathione S-transferase, pi 1
*Gstt1*	2/4 (B6/JF1)	4 to 64	9	10	Glutathione S-transferase, theta 1
*Hexa*	3/4 (B6/JF1)	80 to 111	36	9	Hexosaminidase A (Tay-Sachs Disease)
*Kcnma1*	1/4 (JF1)	8	54	14	Calcium-activated potassium channel
*Thy1*	3/3 (B6/JF1)	14 to 173	535	9	Thy-1 cell surface antigen

† Number of cell lines with monoallelic expression/number of evaluable cell lines. In column 2, the expressed allele(s) are shown in parentheses.

The values shown for columns 2–4 are from Illumina sequencing (see text).

The additional genes analyzed are potentially relevant to diverse genetic and somatic disorders. *Chl1*, “close homologue of L1”, is a cell adhesion gene, and member of the immunoglobulin superfamily. *Chl1* plays a role in neuronal migration and survival [23], and in adult mice, it plays a necessary role in the cycling process of synaptic vesicles [24]. *Chl1* knockout mice show behavioral defects [25], and in human the homologous CALL gene has been implicated in schizophrenia [26]. Although only one cell line, 3A1, showed monoallelic expression for this gene (in the undifferentiated state as well as following differentiation to neurons and astrocytes), its expression pattern warrants further study because of its importance in the CNS.

*Gabrg1* codes for a subunit of the GABA-A subgroup of receptors for the inhibitory neurotransmitter γaminobutyric acid (GABA). It is one of two such genes that has been implicated in alcohol dependence in humans [27], [28]. *Gabrg1* shows monoallelic expression in 2 of 3 evaluable NSC lines (2A1 and 4A5a). Furthermore, RT-PCR results demonstrate allele-specific expression (2A1, 4A5) or a trend (4B3), which is maintained during in vitro differentiation.

The functions of the *Kcnma1* gene are varied, with generalized epilepsy as one of the major phenotypes associated with gene malfunction [29]. Our cSNP-seq analysis shows that the *Kcnma1* gene is expressed from the JF1 allele in cell line 2A1; RT-PCR demonstrates the same pattern in NSC lines 2A5 and 4A5, with a trend toward JF1expression in cell lines 2A1 and 3A1. Monoallelic expression or a strong trend is preserved in these lines as the cells differentiate. The *Kcnma1* gene is large, and alternate splicing has been shown to affect function. We therefore examined in detail the cSNP-seq results for all 7 exons containing evaluable SNPs, and found the same pattern of preference for the JF1 allele.

The annexin family, comprising a group of phospholipid-calcium binding proteins, plays an important role in signal transduction (reviewed in Perretti and D'Acquisto [30]). *Anxa1*, a well-characterized member of the family is a component of the anti-inflammatory pathway, including in the CNS [31]. The enzyme also protects against DNA damage in breast cancer cells [32]. RT-PCR analysis confirms monoallelic expression (B6 allele) in cell lines 2A1 and 2A5, and, in addition, shows expression of the same allele in cell line 4B3. Two of the cell lines (2A1 and 4B3) preserve the same pattern following differentiation to primitive neurons. We also confirmed monoallelic expression of a second member of the annexin gene family, *Anxa2*.

*Hexa* and *Gm2a* code for two interacting protein subunits, mutations of which result in “GM2 gangliosidosis”, Tay-Sachs Disease in the case of Hexa, and “Tay-Sachs-like Disease” in the case of Gm2a. The *Hexa* gene codes for a subunit of β-hexosaminidase A, a lysosomal enzyme involved in the breakdown of gangliosides, while the *Gm2a* gene, codes for a small glycolipid transport protein that is a co-factor for β-hexosaminidase A. Together they catalyze the lysosomal breakdown of the ganglioside GM2 (or other N-acetyl hexosamines). The subunit most commonly mutated is Hexa, resulting in an autosomal recessive disease that affects neurons in the CNS [33]. This inheritance pattern and the relative lack of phenotypic variation in heterozygotes for Tay Sachs Disease suggest that random monoallelic expression does not noticeably influence (human) disease development. However understanding the role of monoallelic expression in regulation of these genes may be important in the ultimate design of gene-based therapies.

*Gm2a* shows monoallelic expression (B6 allele) in three of the cell lines assayed by RT-PCR, with 1 and 2 cell lines maintaining the pattern, in neurons and astrocytes, respectively. The *Hexa* gene shows more robust preservation of the monoallelic pattern. Of the three cell lines showing monoallelic expression, 2A1 and 4A5 express the JF1 allele, while 3A1 expresses the B6 allele. This pattern is maintained in both differentiated cell types except for 4A5, which shows a trend towards JF1 monoallelic expression.

Allele-specific expression of the *Thy1* gene (CD90, thymocyte differentiation antigen 1) in various cells and states of differentiation is of interest because of its potential broad-based functions in apoptosis, axon growth regulation, cell adhesion, inflammation and metastasis [34]. cSNP-seq analysis revealed monoallelic expression of *Thy1* in the three NSC lines in which it was detected (JF1 allele, 2A1 and 4A5a; B6 allele, 2A5). RT-PCR confirmed expression of the JF1 allele in cell line 2A1, and a trend towards expression of the B6 allele in cell line 2A5. RT-PCR also showed expression of the JF1 allele in three additional cell lines, 3A1, 4A5 and 4B3. The pattern is generally maintained following differentiation to astrocytes and neurons (Table S3).

### Random vs. haplotype-dependent monoallelic expression

Figure 2B suggests that haplotype-dependent (i.e., sequence-specific or strain specific) expression may account for a significant proportion of the 152 autosomal genes showing monoallelic expression in at least 2 of 3 evaluable cell lines. Of these genes, 16 genes showed monoallelic expression from the B6 allele only and 59 genes showed expression from the B6 allele or biallelic expression; 28 genes showed expression from the JF1 allele only, while 23 genes showed expression from the JF1 allele or biallelic expression. In addition to the 8 known imprinted genes, 18 genes showed monoallelic expression from both the B6 and JF1 alleles, providing a clear demonstration of haplotype-independence. These are listed in Table S5.

The apparent bias towards expression of the same allele in multiple cell lines is consistent with previous reports of haplotype-dependent monoallelic expression [15], [35]. However, we note that our present data cannot easily distinguish between strain-dependent and random monoallelic expression for the following reasons: 1) The 4 NSC lines we analyzed are derived from the same inbred hybrid mouse strain, yet the majority of genes showing a strain-specific bias also show biallelic expression in some cell lines. This variability between genetically identical cell lines suggests a stochastic component. 2) Our previous results suggest that there may be selection of a small subpopulation of cells in the parental NSC lines prior to generation of clones [7]. This may result in an impression of bias towards one allele across 4 cell lines even in cases of random monoallelic expression. 3) Of the genes we analyzed by RT-PCR, only 3 genes show monoallelic expression from either the B6 or JF1 allele, while 10 genes show bias towards one allele; the pattern is preserved in most cell lines during in vitro differentiation to neurons and glia (Table 1, Table S3). However, there is essentially equal expression from both parental alleles in F_1_ hybrid adult cortex, the tissue from which the NSC lines were derived (Figure S4). These results are not consistent with haplotype-specific expression that extends to mature neurons and/or glia. In conclusion, for most genes, our current results are consistent with strain-specific and/or random monoallelic expression. Future studies should allow us to distinguish between these possibilities.

### Perspective: Relevance of Results to Human Disease

We report here the first transcriptome-wide direct analysis of monoallelic expression in clonal cells of neural origin. We have previously noted the potential connection between inheritance patterns of a number of major human disorders and monoallelic expression of relevant genes [6], [7]. For example, the diseases associated with *Chl1* (schizophrenia), *Gabrg1* (alcohol dependence) and *Kcnma1* (epilepsy) all display twin discordance consistent with random monoallelic expression. In addition, Table 2 illustrates a potential link between genes that show allele-specific expression and signal transduction, mutagenesis, and the response to stress and inflammation. Consistent with these functions, there have been numerous studies linking changes in expression of glutathione transferases, particularly *Gspt1* and annexin genes to various cancers (reviewed in Tew et al. [36] and Mussunoor and Murray [37]). In these cases, heritable monoallelic expression in a given clone of cells may be considered as the equivalent of loss of heterozygosity. In this scenario, mutation of the one active allele of a tumor suppressor or potential oncogene might lead to rapid carcinogenesis, depending upon the pathway.

## Methods

Detailed experimental procedures are in Supporting Information. The JF1 cSNP library is available at the dbSNP database (www.ncbi.nlm.nih.gov). Results at each cSNP for the 4 cell lines analyzed by Illumina sequencing are available online at http://genome.ucsc.edu.

### Ethics statement

Procedures on mice involved little or no pain or distress and were approved by the Institutional Animal Care and Use Committee at the City of Hope (IACUC #97013, approved until 12/20/2011.) City of Hope is accredited by AAALAC.

### Mice, cell lines and cSNP-seq analysis

B6×JF1 hybrid mice and the clonal NSC lines 2A1, 2A5, 3A1, 4A5 and 4B3 have been previously described [7]. NSC line 4A5a is a subclone of 4A5. Next-generation sequencing was carried out by the City of Hope DNA sequencing/Solexa Core. Details of sequencing, alignment and data analysis are in Methods S1.

### RT-PCR

Conditions of RT-PCR, and quantitative automated sequencing of amplified products were previously described [7]. Primers used are listed in Table S4. Differentiation of NSC lines to astrocytes and neurons was previously described [6].

## Supporting Information

Figure S1 cSNP-seq analysis of JF1 and NSC transcripts. (A) Short reads sequenced and mapped with different filtering criteria. (B) Lower-left panels: scatter plots of log2-transformed RPKM for JF1 and 4 hybrid cell lines. Lowess smoothing is used to draw the lines. Upper-right panels: Calculated Pearson correlation among the samples. *** indicates the correlations are significant at *p*-value<0.001 from a t-test. (C) Distribution of JF1 SNP depth of coverage for 118,579 cSNPs identified on 12,416 Refseq genes. SNPs with less than 3 or larger than 2000 coverage have been removed. (D) Chromosomal distribution of Refseq genes with monoallelic expression in the 4 NSC lines.
(PDF)

Figure S2 Quantile plot for Refseq genes with biallelic or monoallelic expression. (A) 2A1, (B) 2A5, (C) 3A1 and (D) 4A5a. In each case, the log2(RPKM) for genes with biallelic expression is consistently higher than for genes with monoallelic expession up to the 80^th^ to 90^th^ quantile. We observe an approximately 0.5 reduction of log2(RPKM) for genes with monoallelic expression compared to genes with biallelic expression (∼4.0 log2(RPKM) *vs.* ∼4.5 log2(RPKM)). This equals about a 30% reduction in raw RPKM, i.e. transcript levels, for genes with monoallelic expression. The result is statistically significant for all 4 hybrid cell lines, the largest *p*-value being 0.0002 (two sample t-test) for 2A5.
(PDF)

Figure S3 Chromosomal location of autosomal genes with monoallelic expression in NSC lines. The cell lines are indicated in panels A–D.
(PDF)

Figure S4 RT-PCR results for selected genes with monoallelic expression. RT-PCR primers were selected to include a B6/JF1 SNP within the amplified product. For each gene, the first row shows the results of automated sequencing of RNA from brain tissue of B6 and JF1 mice and F1 hybrid progeny, as well as DNA of selected NSC lines. The following 3 rows show results for the NSC lines indicated, both for undifferentiated NSCs and following differentiation to astrocytes or neurons. In each case, the % expression of the predominant allele is shown. Technical replicates are in parentheses. For *n* = 2, the range is shown; for *n* = 3, the SEM is given. A1, *Anxa1*; A2, *Anxa2*; B, *Chl1*; C, *Gabrg1*; D, *Gm2a*; E, *Gstk1*; F, *Gstm5*; G, *Gsto1*; H, *Gstp1*; I, *Gstt1*; J, *Hexa*; K, *Kcnma1*; L, *Thy1*.
(PDF)

Table S1 Number of Refseq genes in each category of allele-specific expression by cell line.
(DOC)

Table S2 Enrichment of autosomal gene clusters showing monoallelic expression by use of the DAVID Bioinformatics Site.
(DOC)

Table S3 Allele-specific expression of selected genes in NSC lines before and after differentiation to astrocytes or neurons.
(DOC)

Table S4 List of primers.
(DOC)

Table S5 Genes with random (i.e., haplotype-independent) monoallelic expression detected by RNA-seq.
(DOC)

Methods S1 Details of Illumina sequencing, alignment and data analysis.
(DOC)

Dataset S1 Expression and classification of monoallelic expression for genes evaluable in NSC cell lines 2A1, 2A5, 3A1 and 4A5a.
(XLS)
