# Supplementary figures and images for: Transcriptome-Wide Survey of Mouse CNS-Derived Cells Reveals Monoallelic Expression within Novel Gene Families
Source: PLoS One. 2012 Feb 22;7(2):e31751. doi: 10.1371/journal.pone.0031751 (PMC3285176; doi:10.1371/journal.pone.0031751)

A

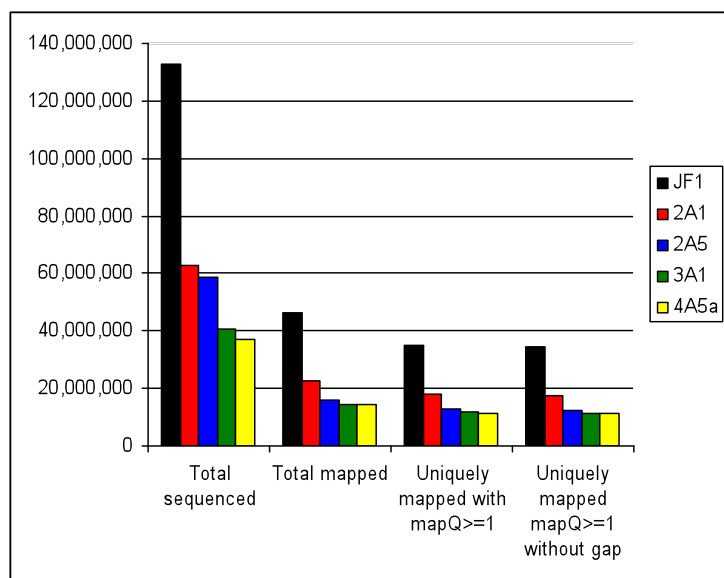

B

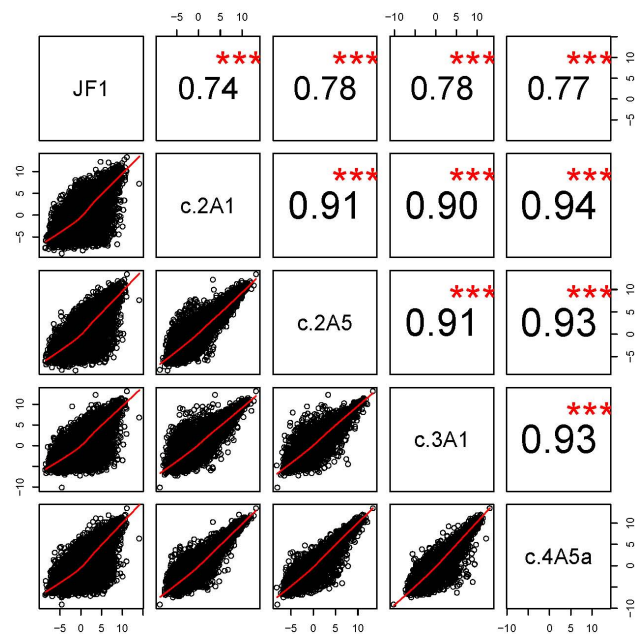

C

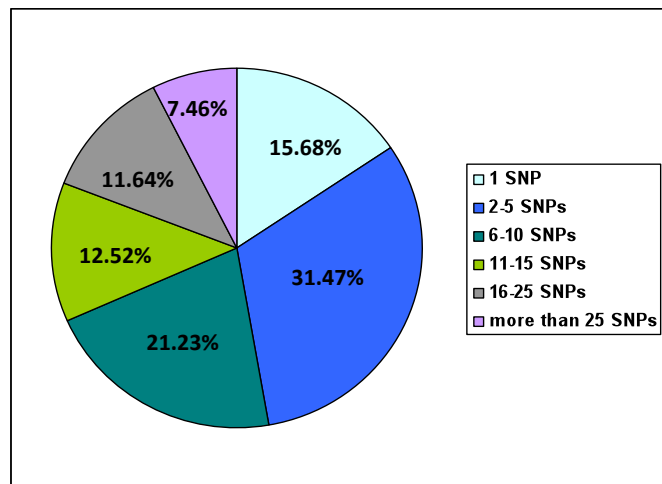

D

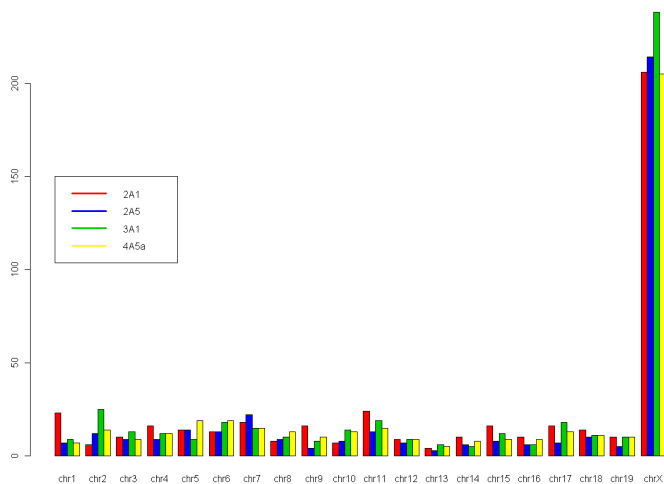

Supplement: Figure S1 — cSNP-seq analysis of JF1 and NSC transcripts. (A) Short reads sequenced and mapped with different filtering criteria. (B) Lower-left panels: scatter plots of log2-transformed RPKM for JF1 and 4 hybrid cell lines. Lowess smoothing is used to draw the lines. Upper-right panels: Calculated Pearson correlation among the samples. *** indicates the correlations are significant at p-value<0.001 from a t-test. (C) Distribution of JF1 SNP depth of coverage for 118,579 cSNPs identified on 12,416 Refseq genes. SNPs with less than 3 or larger than 2000 coverage have been removed. (D) Chromosomal distribution of Refseq genes with monoallelic expression in the 4 NSC lines. (PDF) [file pone.0031751.s001.pdf]

A: 2A1

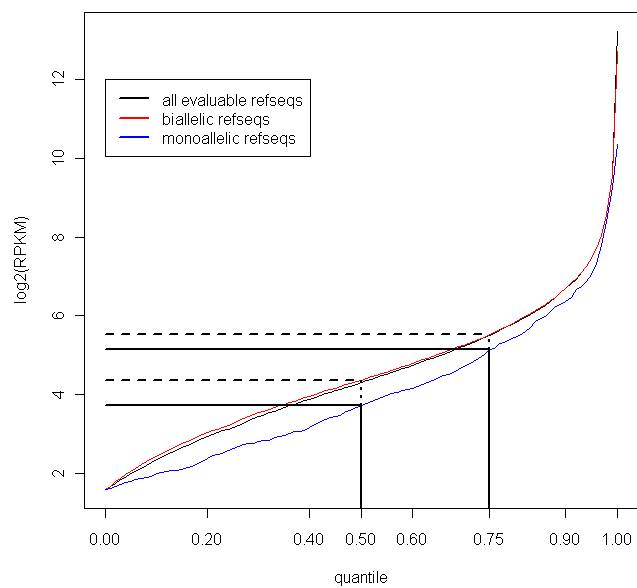

B: 2A5

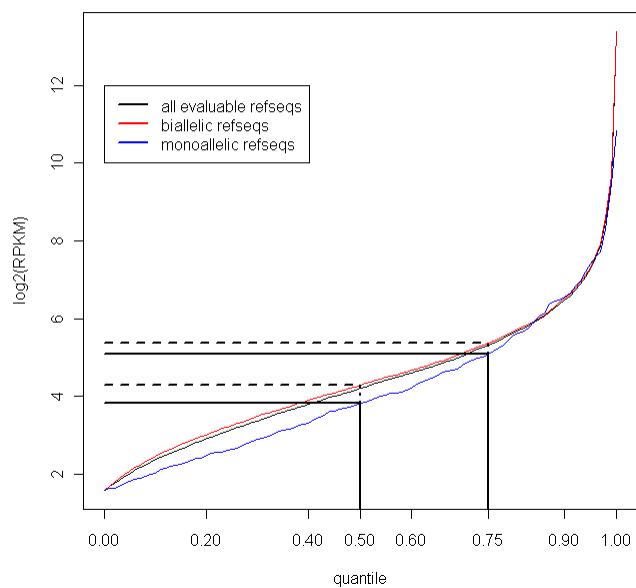

C: 3A1

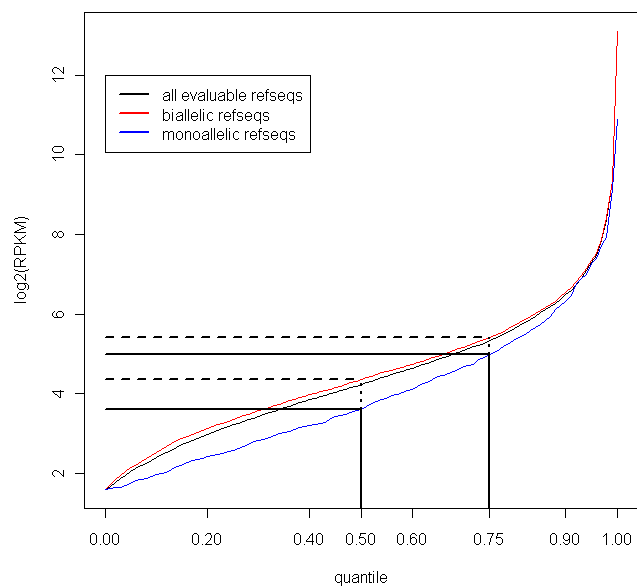

C: 4A5a

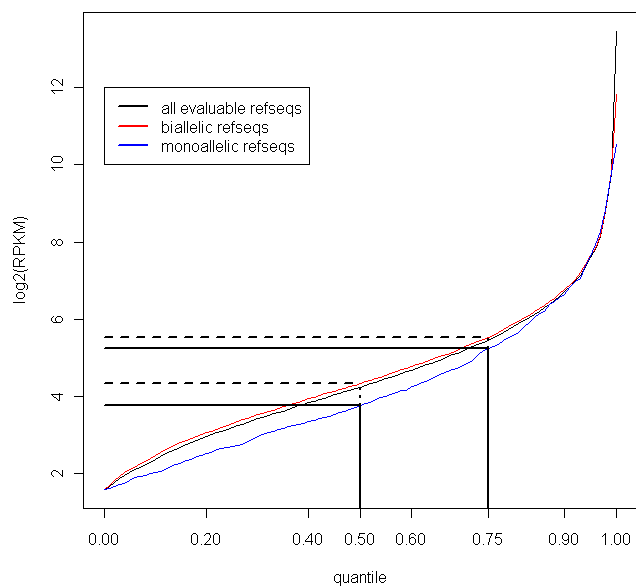

Supplement: Figure S2 — Quantile plot for Refseq genes with biallelic or monoallelic expression. (A) 2A1, (B) 2A5, (C) 3A1 and (D) 4A5a. In each case, the log2(RPKM) for genes with biallelic expression is consistently higher than for genes with monoallelic expession up to the 80th to 90th quantile. We observe an approximately 0.5 reduction of log2(RPKM) for genes with monoallelic expression compared to genes with biallelic expression (∼4.0 log2(RPKM) vs. ∼4.5 log2(RPKM)). This equals about a 30% reduction in raw RPKM, i.e. transcript levels, for genes with monoallelic expression. The result is statistically significant for all 4 hybrid cell lines, the largest p-value being 0.0002 (two sample t-test) for 2A5. (PDF) [file pone.0031751.s002.pdf]

A: 2A1

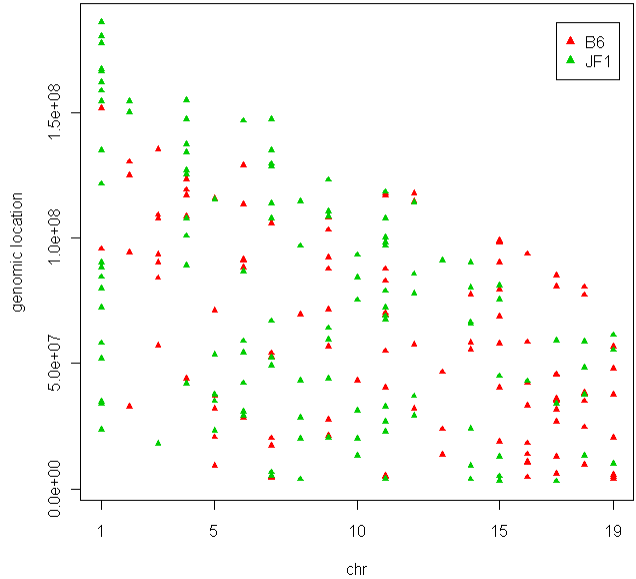

B: 2A5

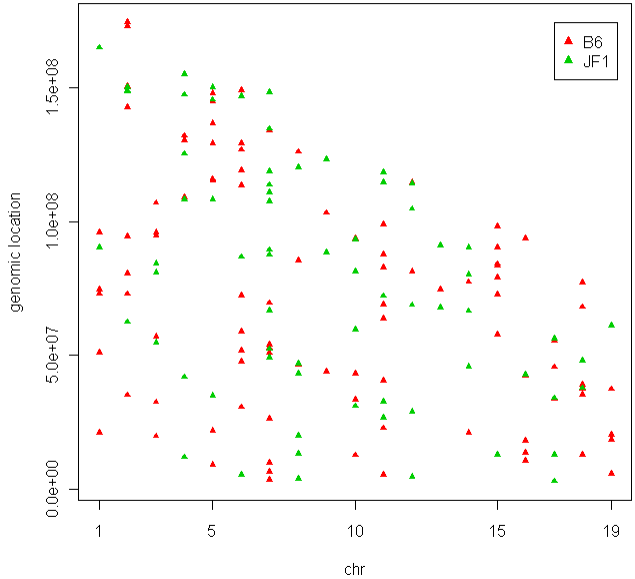

C: 3A1

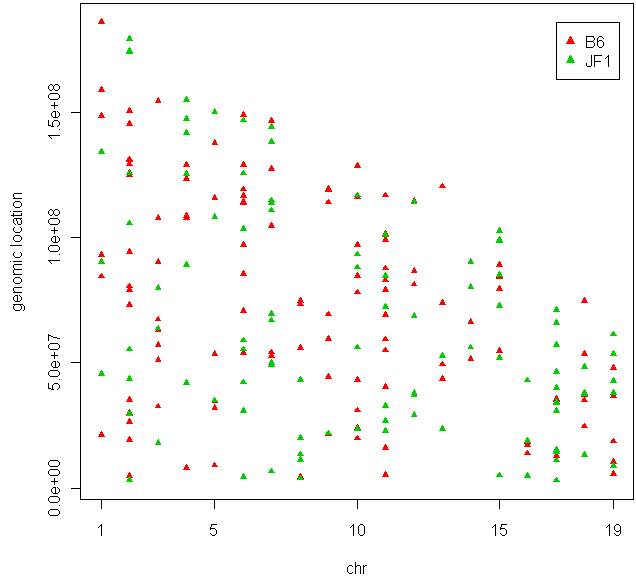

D: 4A5a

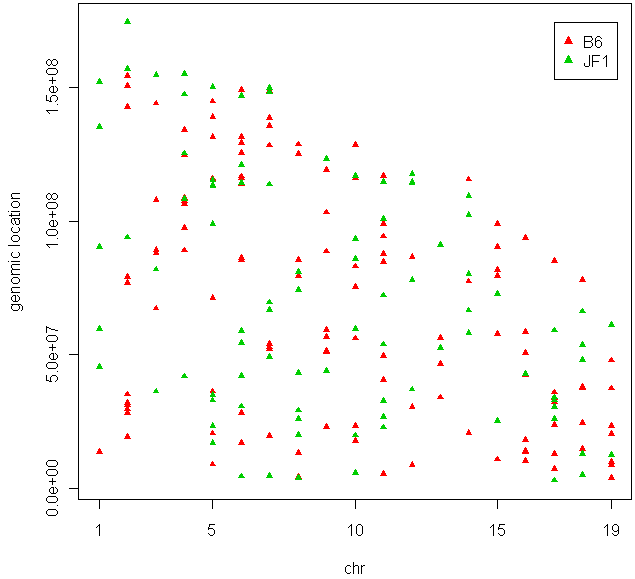

Supplement: Figure S3 — Chromosomal location of autosomal genes with monoallelic expression in NSC lines. The cell lines are indicated in panels A–D. (PDF) [file pone.0031751.s003.pdf]
