# Supplementary material for: Transcriptome-Wide Survey of Mouse CNS-Derived Cells Reveals Monoallelic Expression within Novel Gene Families
Source: PLoS One. 2012 Feb 22;7(2):e31751. doi: 10.1371/journal.pone.0031751 (PMC3285176; doi:10.1371/journal.pone.0031751)
Supplement: Figure S4 — RT-PCR results for selected genes with monoallelic expression. RT-PCR primers were selected to include a B6/JF1 SNP within the amplified product. For each gene, the first row shows the results of automated sequencing of RNA from brain tissue of B6 and JF1 mice and F1 hybrid progeny, as well as DNA of selected NSC lines. The following 3 rows show results for the NSC lines indicated, both for undifferentiated NSCs and following differentiation to astrocytes or neurons. In each case, the % expression of the predominant allele is shown. Technical replicates are in parentheses. For n = 2, the range is shown; for n = 3, the SEM is given. A1, Anxa1; A2, Anxa2; B, Chl1; C, Gabrg1; D, Gm2a; E, Gstk1; F, Gstm5; G, Gsto1; H, Gstp1; I, Gstt1; J, Hexa; K, Kcnma1; L, Thy1. (PDF) [file pone.0031751.s004.pdf]

A1

Anxa1

controls

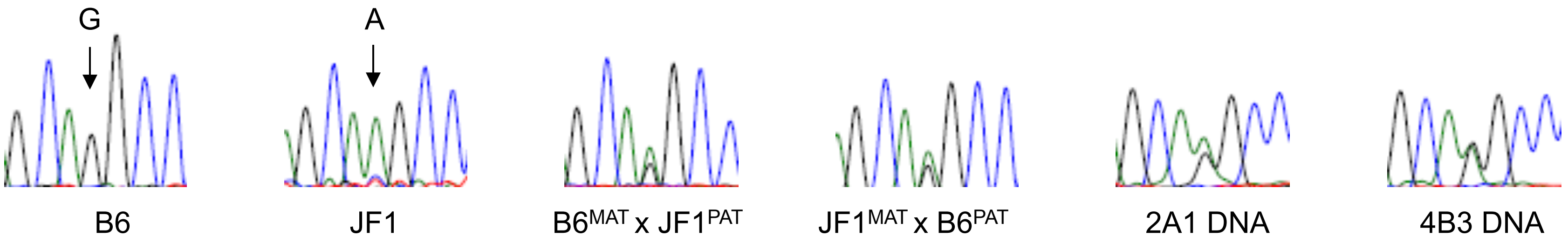

undifferentiated NSCs

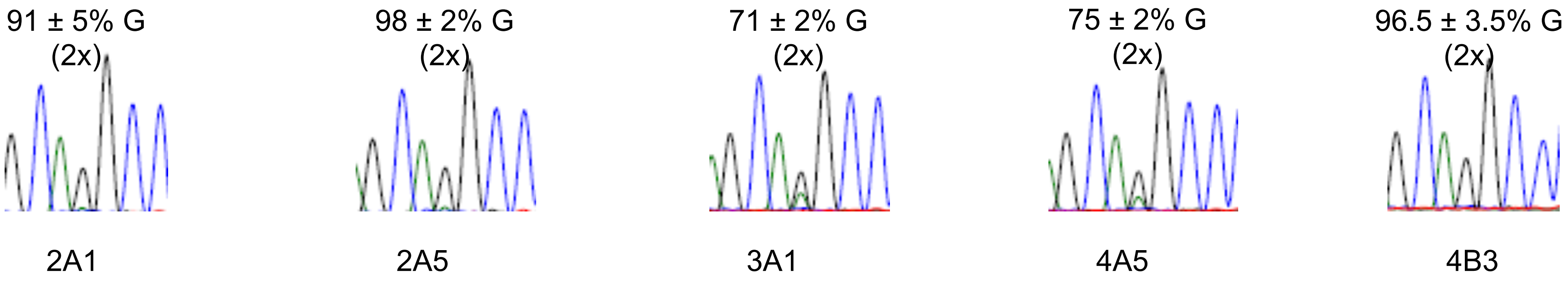

A1

Anxa1 (cont.)

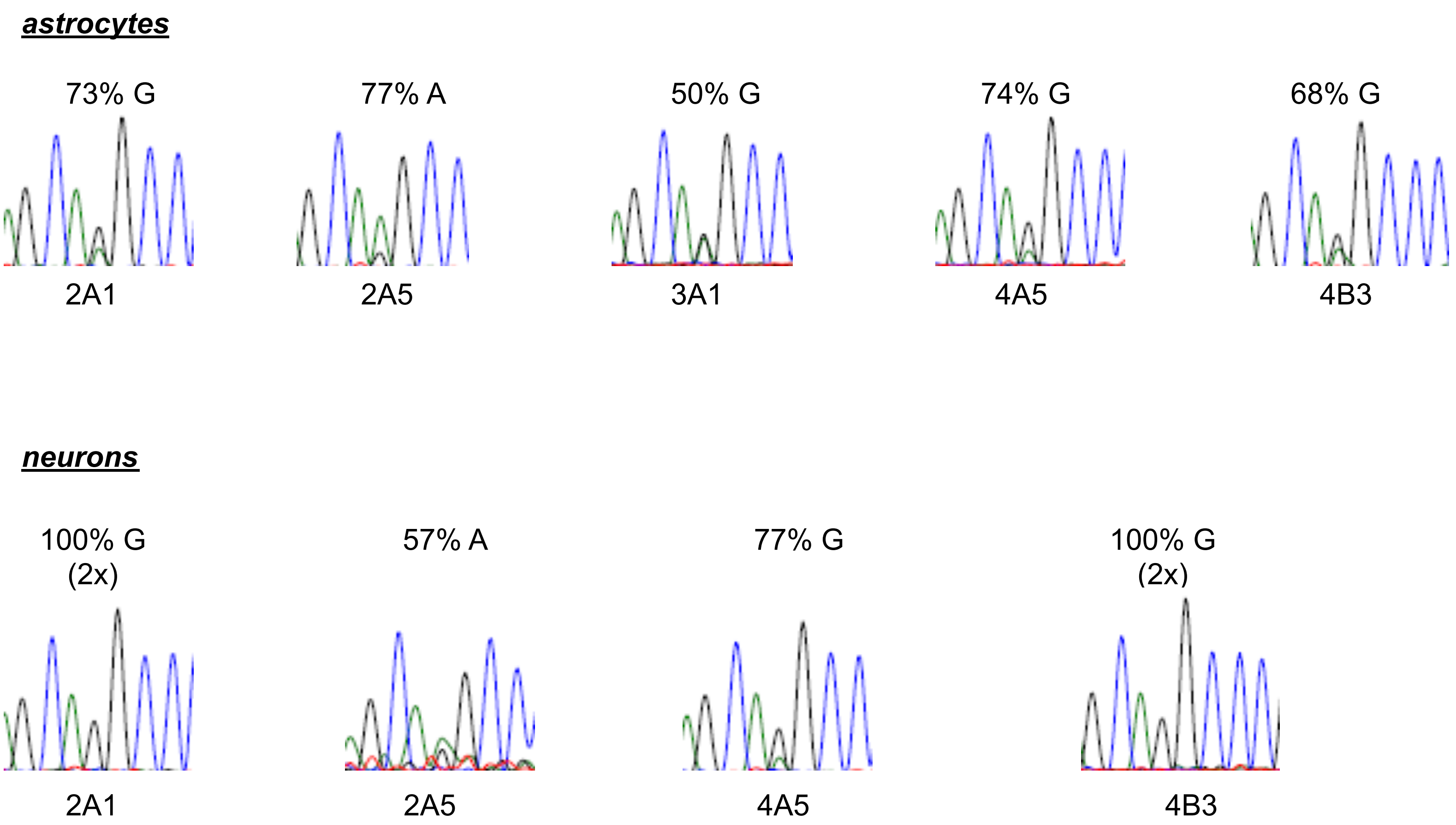

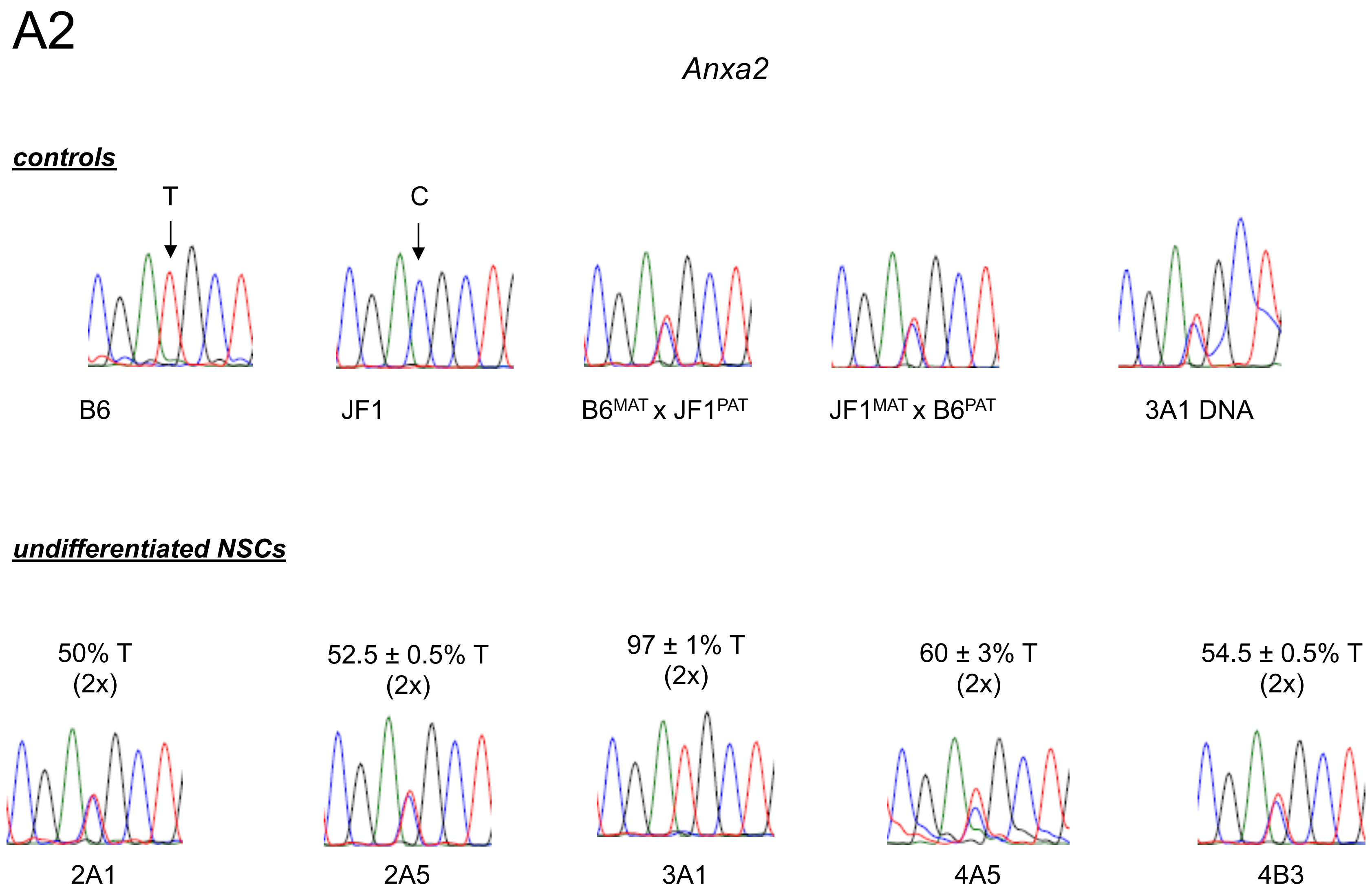
A2
Anxa2
controls
T
C
B6
JF1
B6MAT x JF1PAT
JF1MAT x B6PAT
3A1 DNA
undifferentiated NSCs
50% T
(2x)
52.5 ± 0.5% T
(2x)
97 ± 1% T
(2x)
60 ± 3% T
(2x)
54.5 ± 0.5% T
(2x)
2A1
2A5
3A1
4A5
4B3

A2

Anxa2 (cont.)

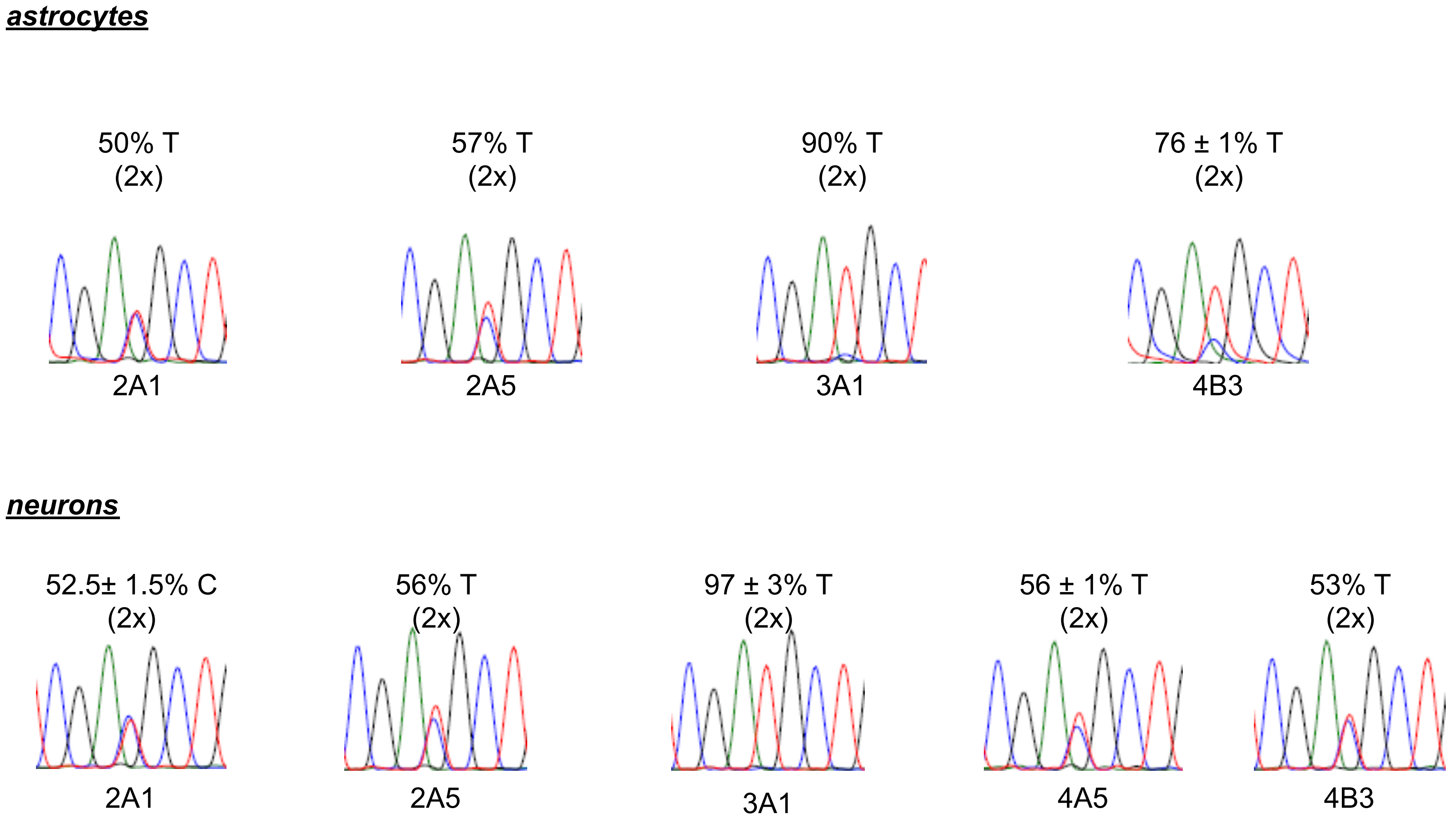

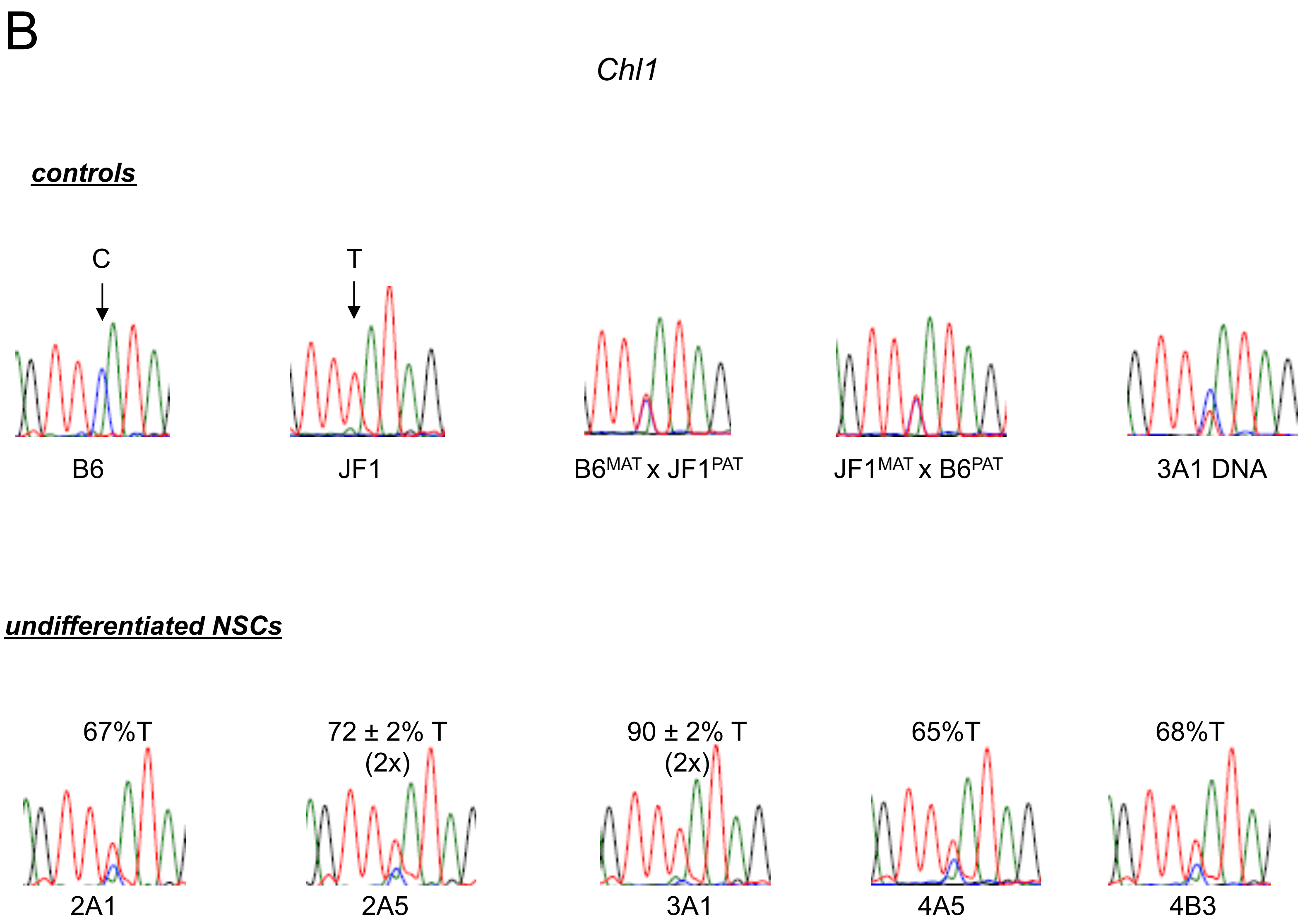
B
Chl1
controls
C
T
B6
JF1
B6MAT x JF1PAT
JF1MAT x B6PAT
3A1 DNA
undifferentiated NSCs
67%T
72 ± 2% T
(2x)
90 ± 2% T
(2x)
65%T
68%T
2A1
2A5
3A1
4A5
4B3

B

Chl1 (cont.)

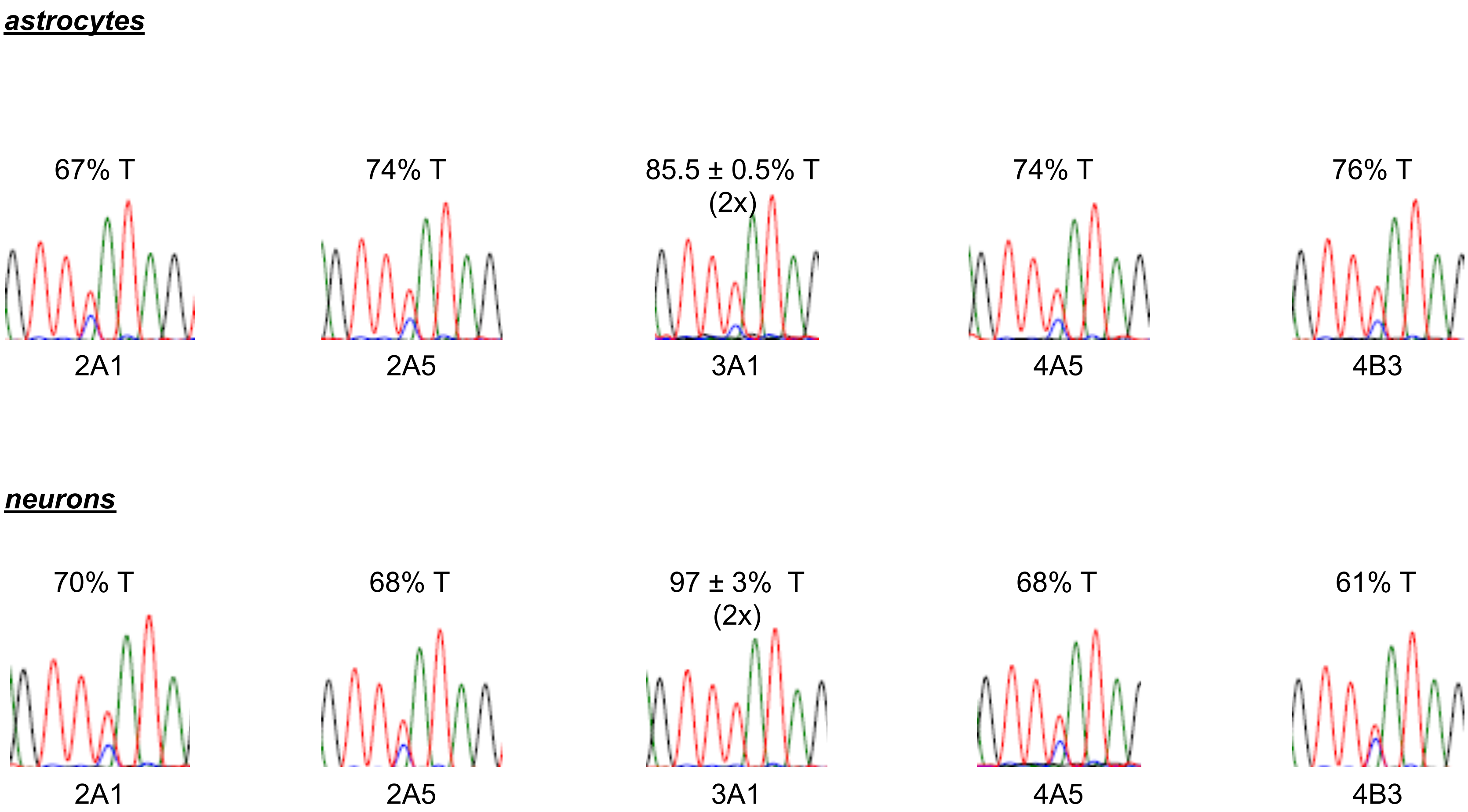
astrocytes
67% T
74% T
85.5 ± 0.5% T
(2x)
74% T
76% T
2A1
2A5
3A1
4A5
4B3
neurons
70% T
68% T
97 ± 3% T
(2x)
68% T
61% T
2A1
2A5
3A1
4A5
4B3

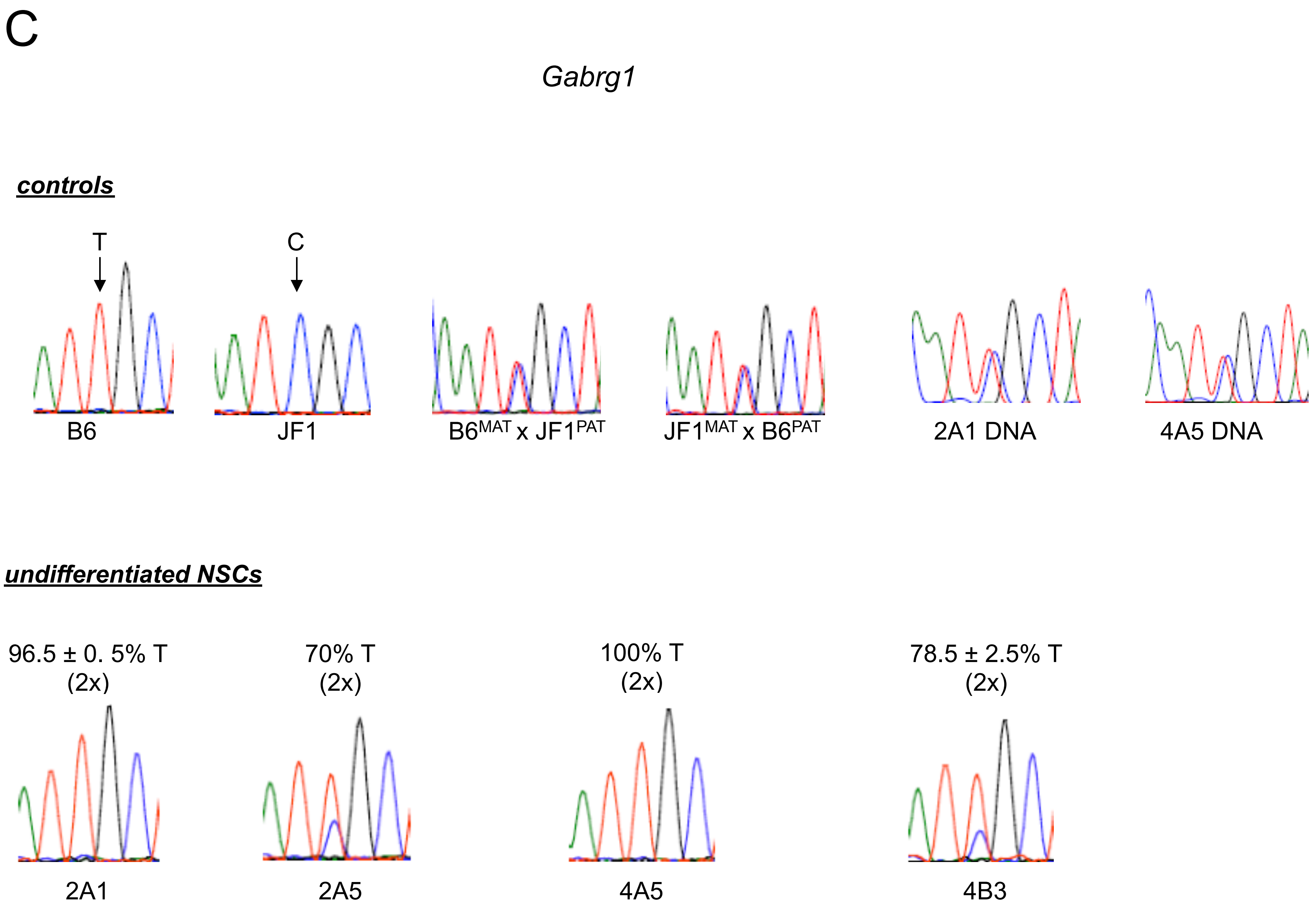
C
Gabrg1
controls
T
C
B6
JF1
B6MAT x JF1PAT
JF1MAT x B6PAT
2A1 DNA
4A5 DNA
undifferentiated NSCs
96.5 ± 0. 5% T
(2x)
70% T
(2x)
100% T
(2x)
78.5 ± 2.5% T
(2x)
2A1
2A5
4A5
4B3

C

Gabrg1 (cont.)

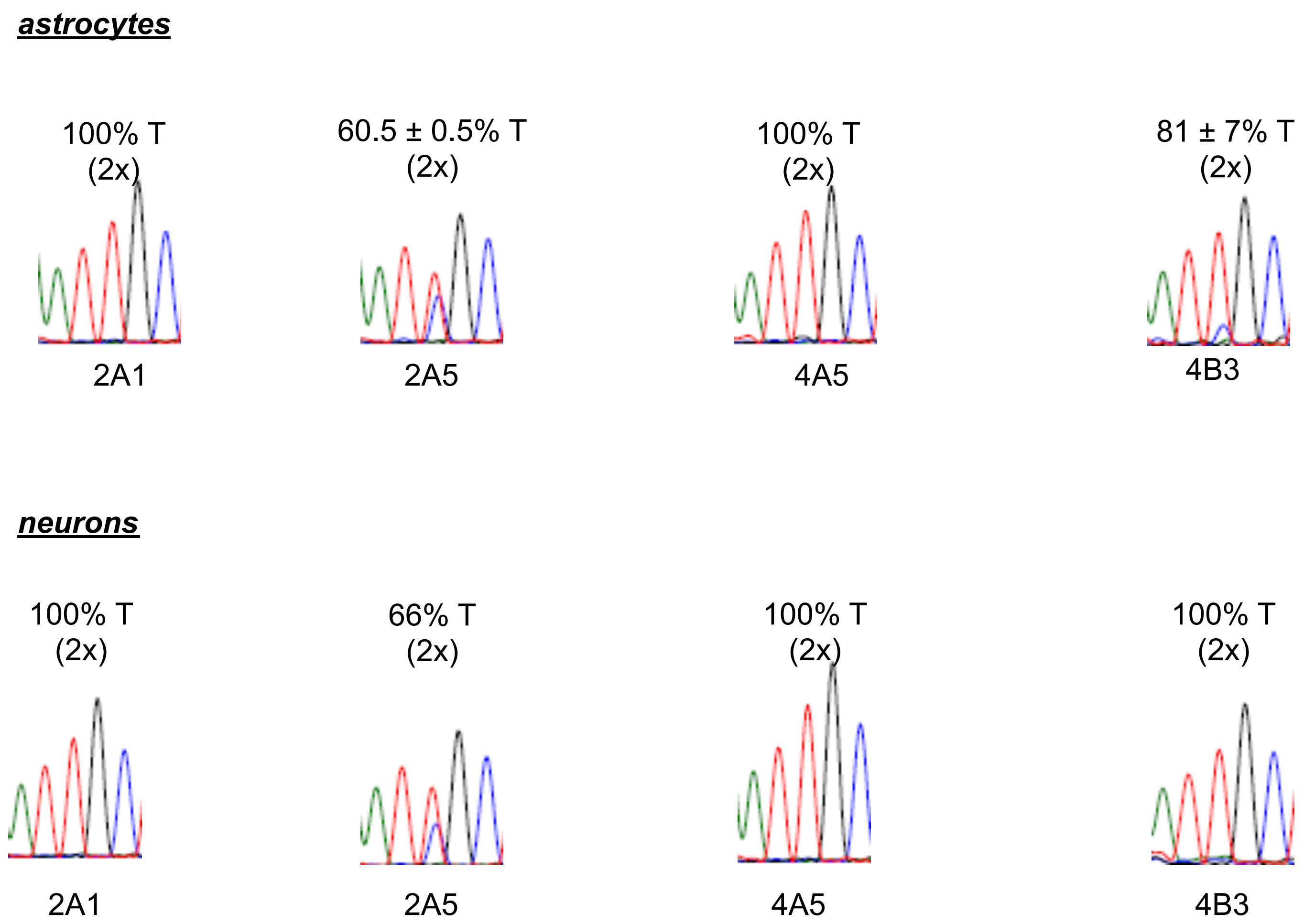

D

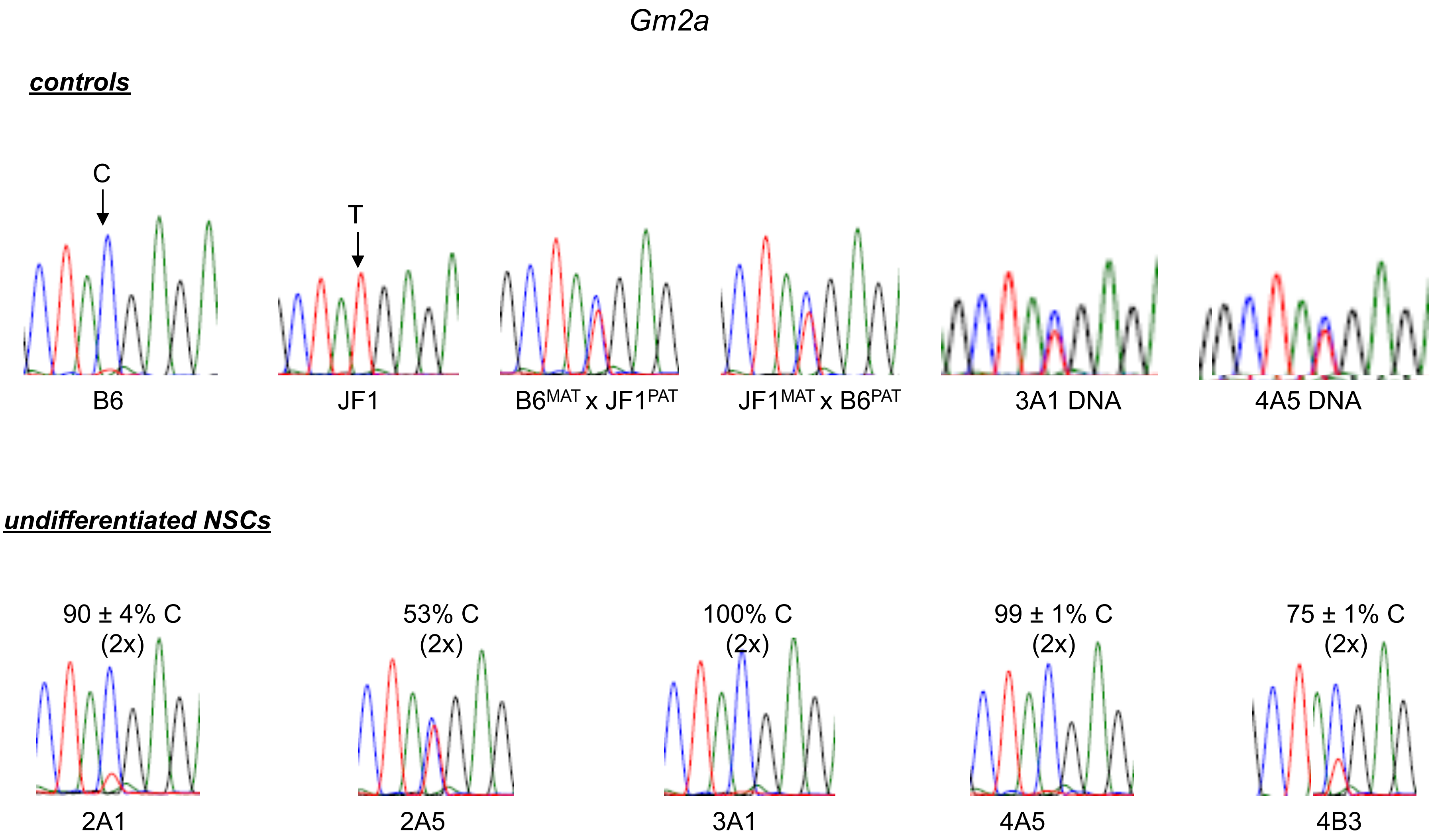

Gm2a
controls
C
T
B6
JF1
B6MAT x JF1PAT
JF1MAT x B6PAT
3A1 DNA
4A5 DNA
undifferentiated NSCs
90 ± 4% C
(2x)
53% C
(2x)
100% C
(2x)
99 ± 1% C
(2x)
75 ± 1% C
(2x)
2A1
2A5
3A1
4A5
4B3

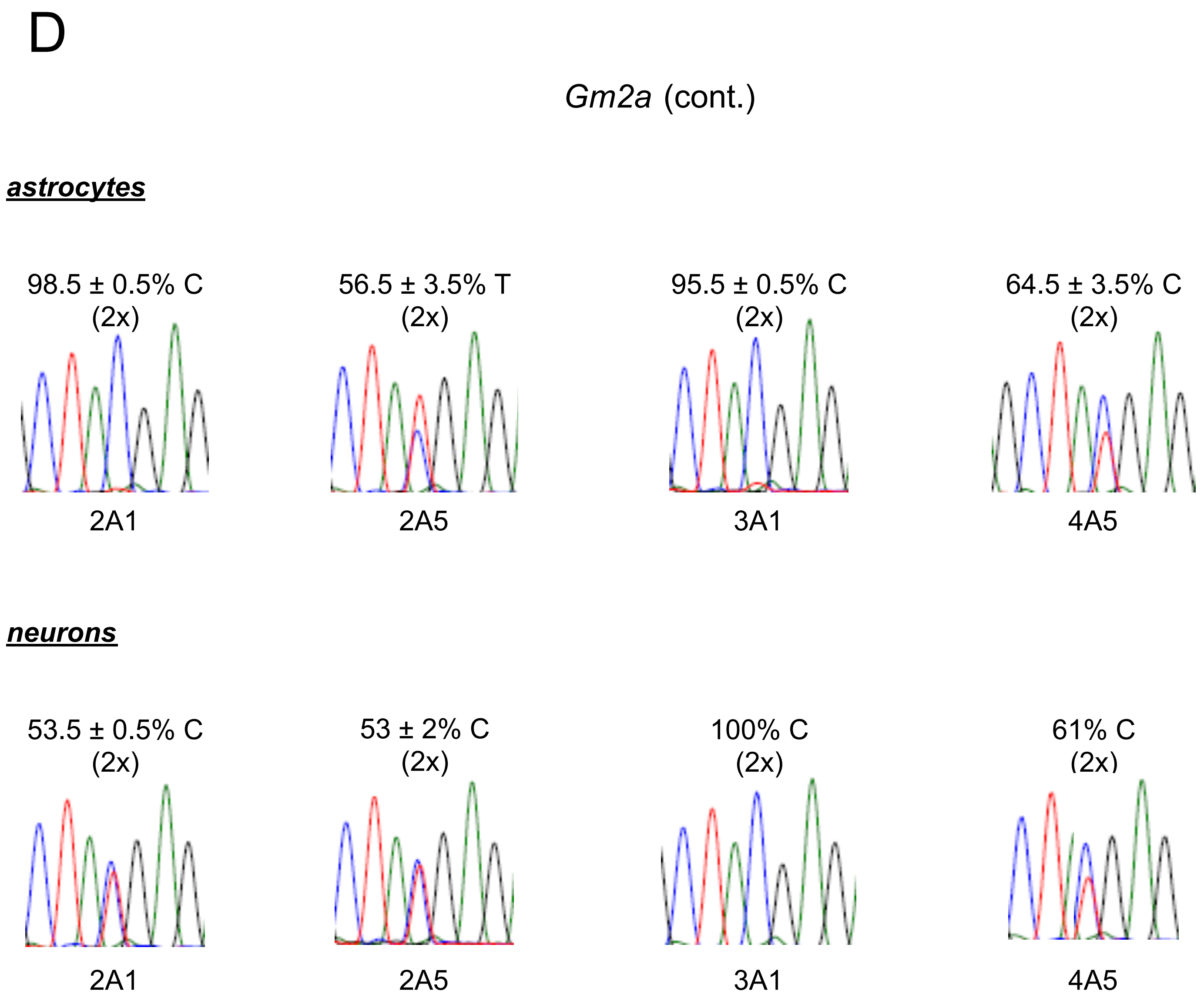
D
Gm2a (cont.)
astrocytes
98.5 ± 0.5% C
(2x)
2A1
56.5 ± 3.5% T
(2x)
2A5
95.5 ± 0.5% C
(2x)
3A1
64.5 ± 3.5% C
(2x)
4A5
neurons
53.5 ± 0.5% C
(2x)
2A1
53 ± 2% C
(2x)
2A5
100% C
(2x)
3A1
61% C
(2x)
4A5

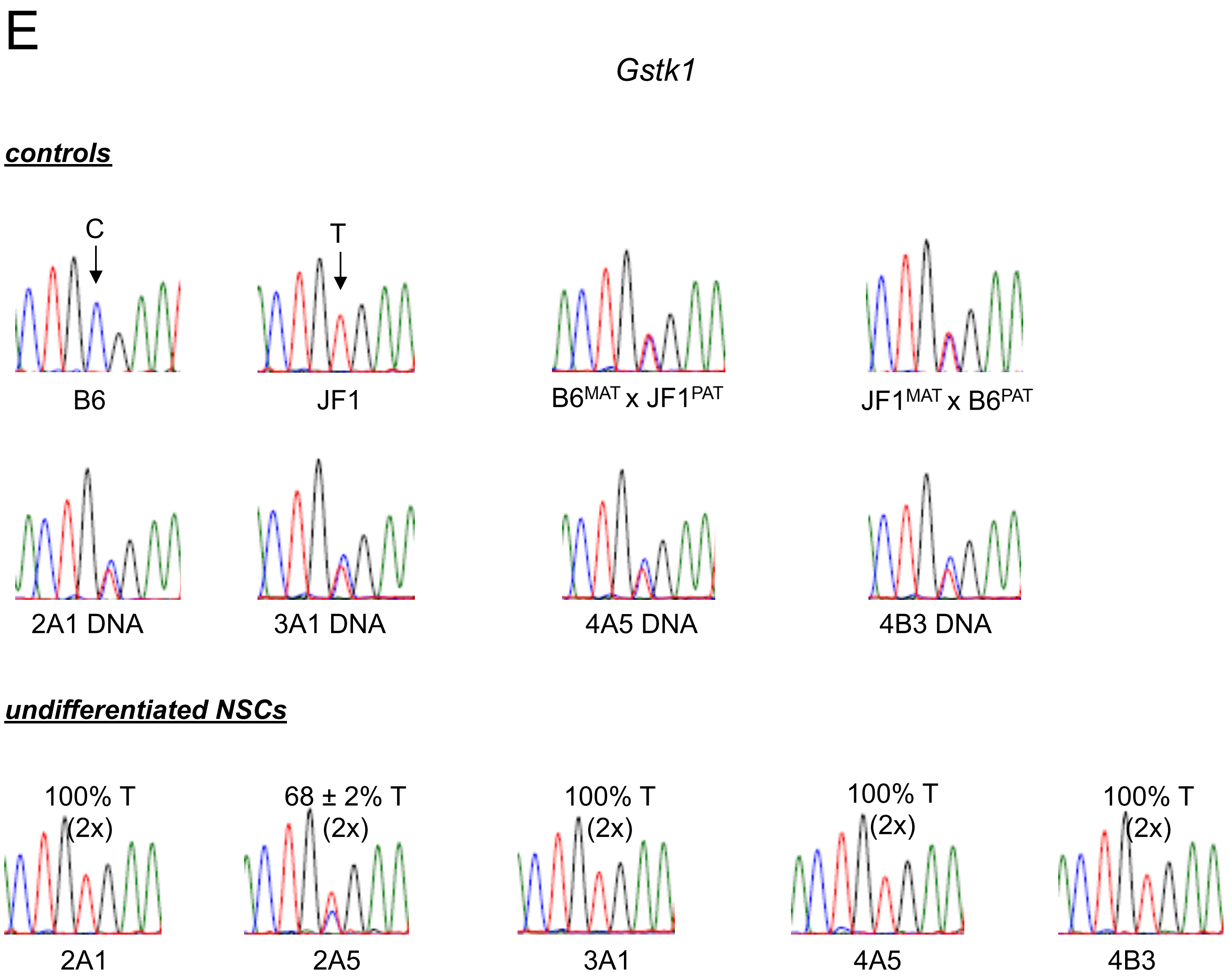

E
Gstk1
controls
C
T
B6
JF1
B6MAT x JF1PAT
JF1MAT x B6PAT
2A1 DNA
3A1 DNA
4A5 DNA
4B3 DNA
undifferentiated NSCs
100% T (2x)
68 ± 2% T (2x)
100% T (2x)
100% T (2x)
100% T (2x)
2A1
2A5
3A1
4A5
4B3

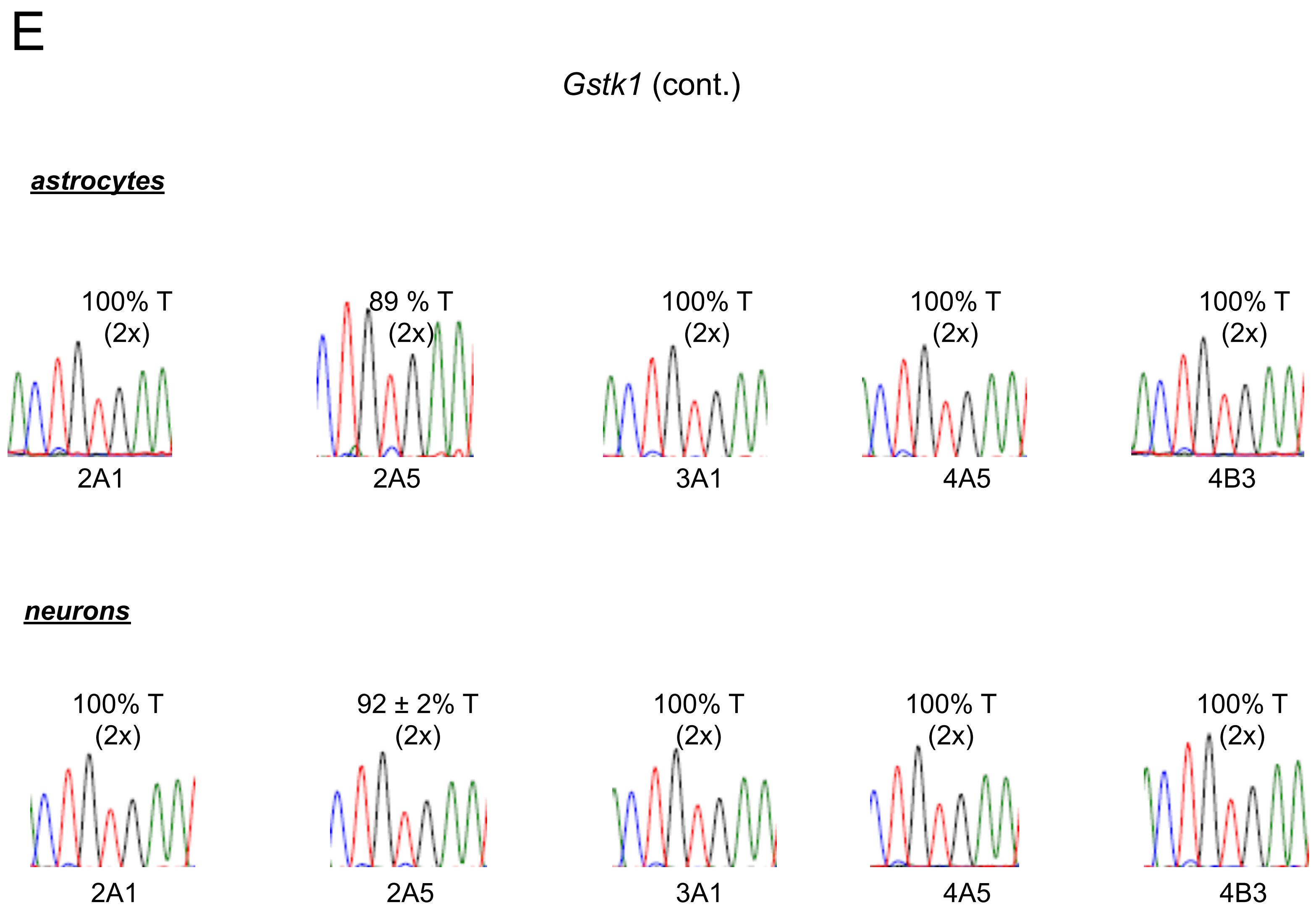
E
Gstk1 (cont.)
astrocytes
100% T (2x)
2A1
89 % T (2x)
2A5
100% T (2x)
3A1
100% T (2x)
4A5
100% T (2x)
4B3
neurons
100% T (2x)
2A1
92 ± 2% T (2x)
2A5
100% T (2x)
3A1
100% T (2x)
4A5
100% T (2x)
4B3

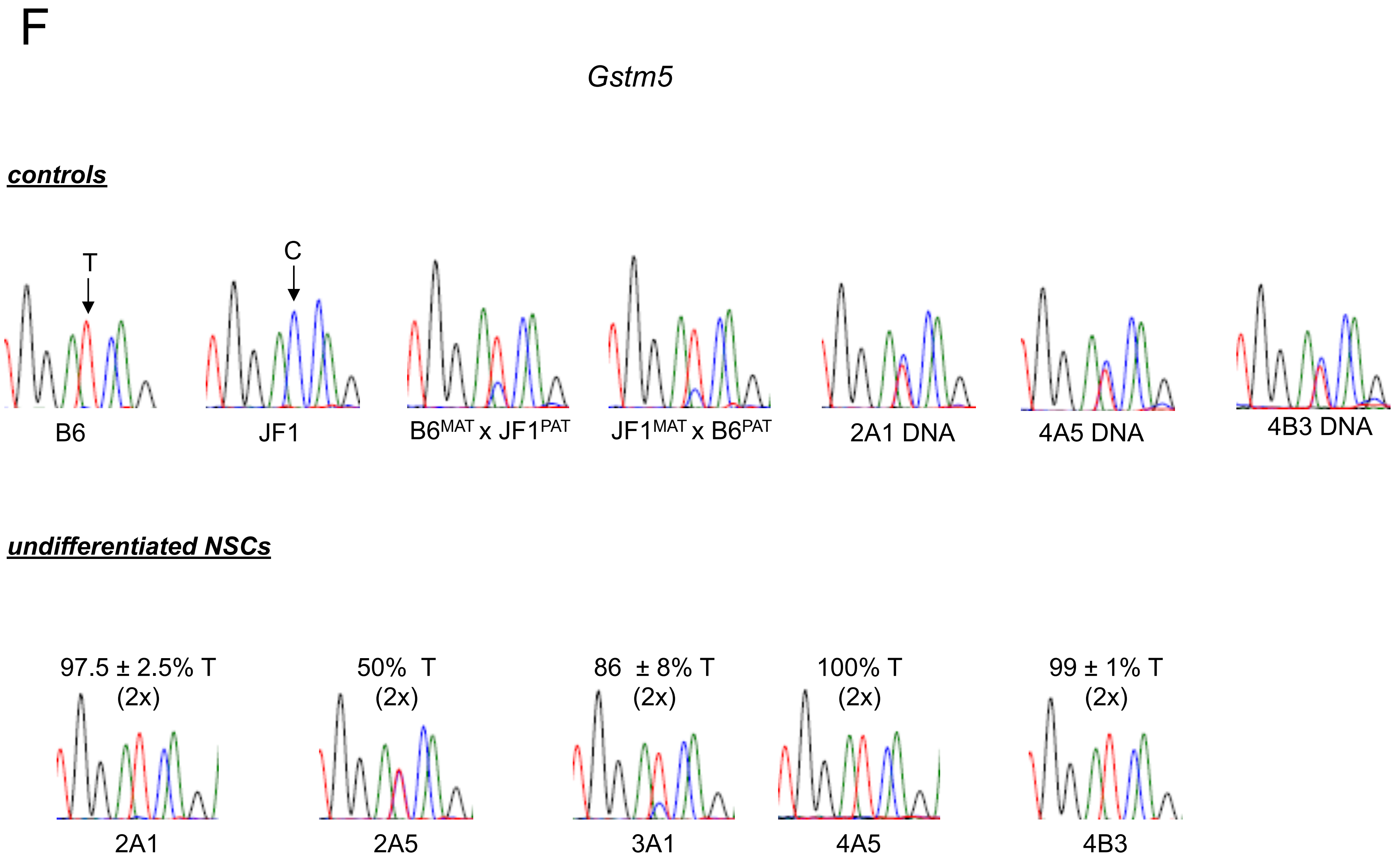
F
Gstm5
controls
T
C
B6
JF1
B6MAT x JF1PAT
JF1MAT x B6PAT
2A1 DNA
4A5 DNA
4B3 DNA
undifferentiated NSCs
97.5 ± 2.5% T
(2x)
2A1
50% T
(2x)
2A5
86 ± 8% T
(2x)
3A1
100% T
(2x)
4A5
99 ± 1% T
(2x)
4B3

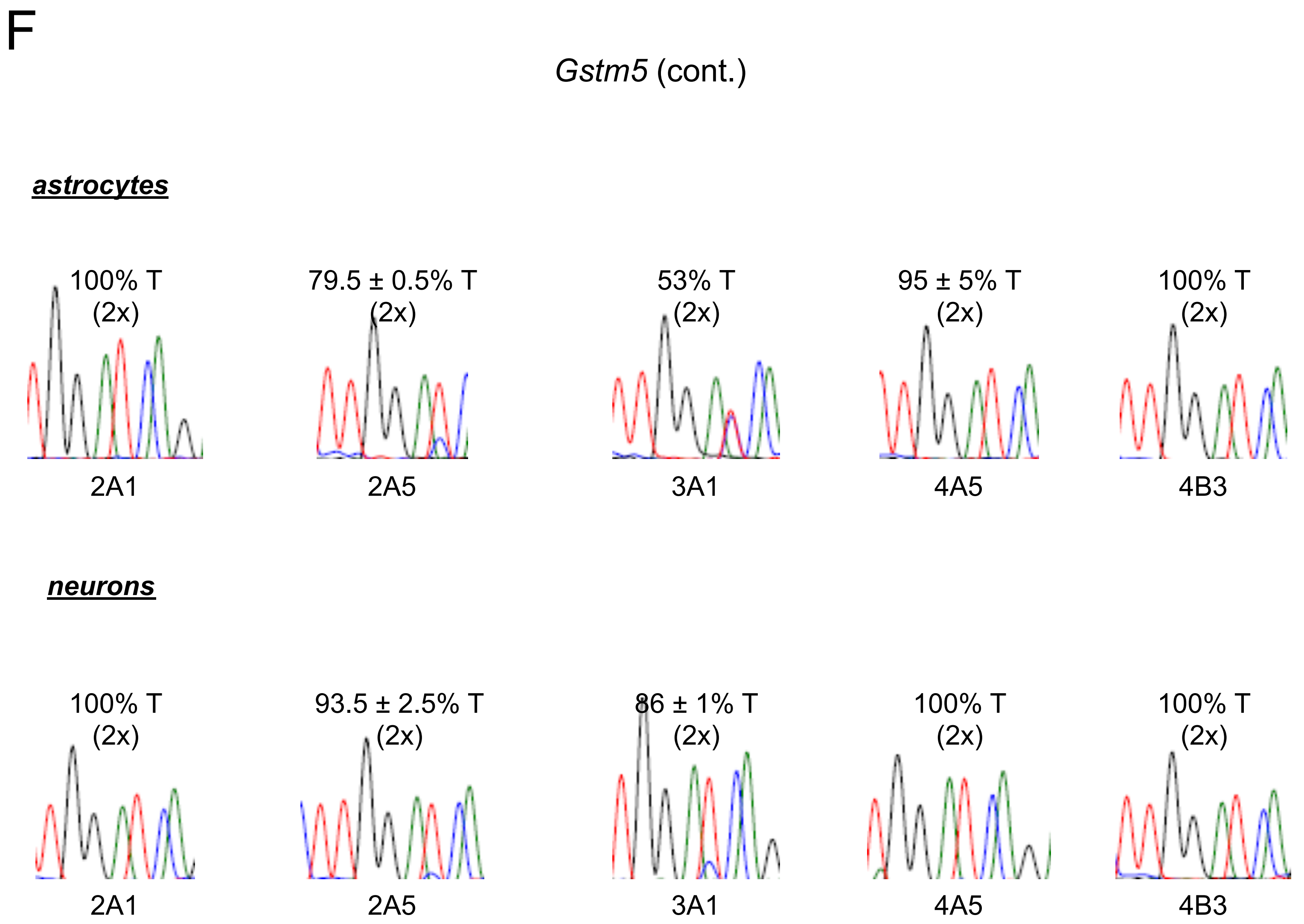
F
Gstm5 (cont.)
astrocytes
100% T
(2x)
2A1
79.5 ± 0.5% T
(2x)
2A5
53% T
(2x)
3A1
95 ± 5% T
(2x)
4A5
100% T
(2x)
4B3
neurons
100% T
(2x)
2A1
93.5 ± 2.5% T
(2x)
2A5
86 ± 1% T
(2x)
3A1
100% T
(2x)
4A5
100% T
(2x)
4B3

G

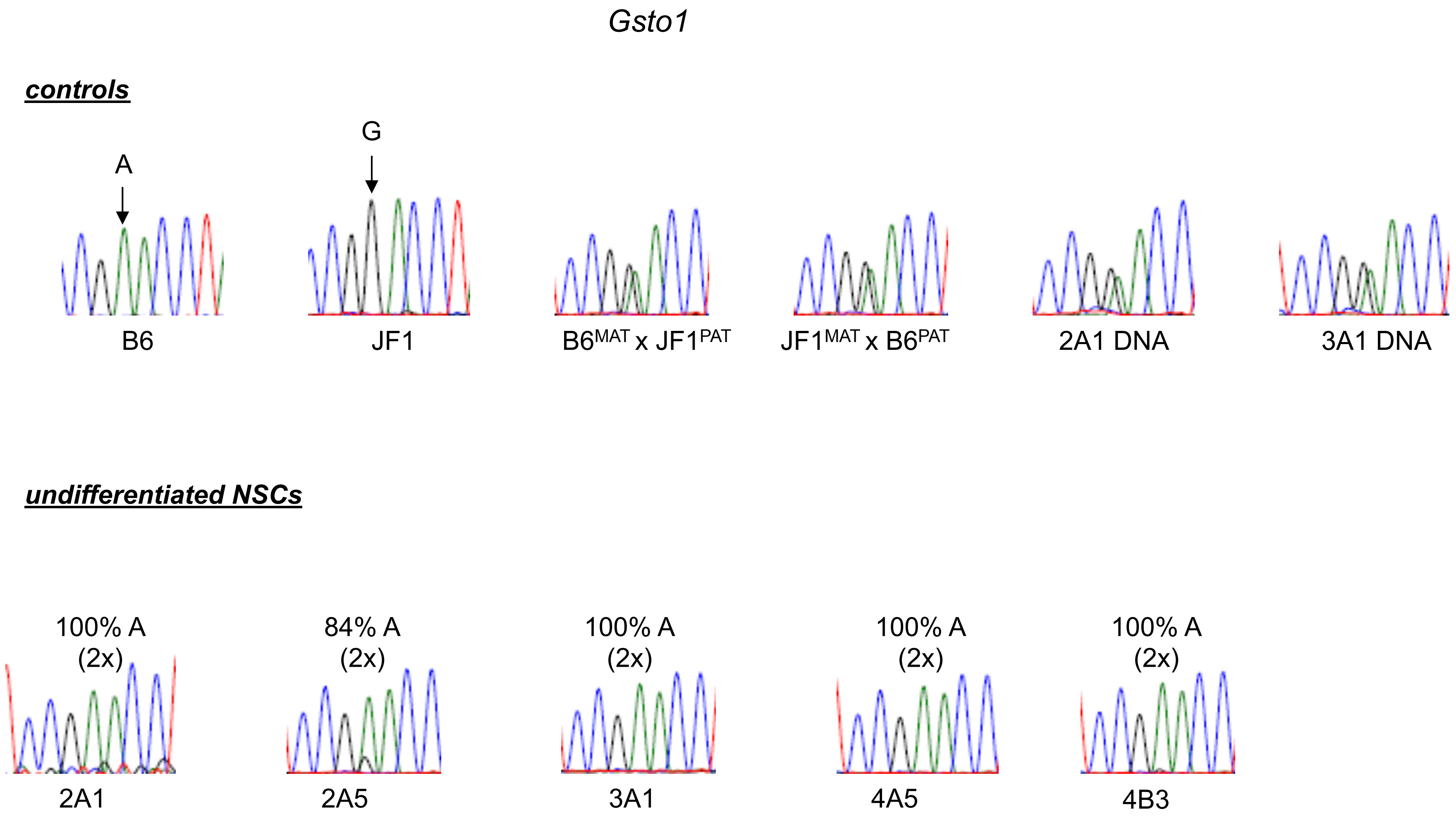

Gsto1
controls
A
G
B6
JF1
B6MAT x JF1PAT
JF1MAT x B6PAT
2A1 DNA
3A1 DNA
undifferentiated NSCs
100% A (2x)
84% A (2x)
100% A (2x)
100% A (2x)
100% A (2x)
2A1
2A5
3A1
4A5
4B3

G

Gsto1 (cont.)

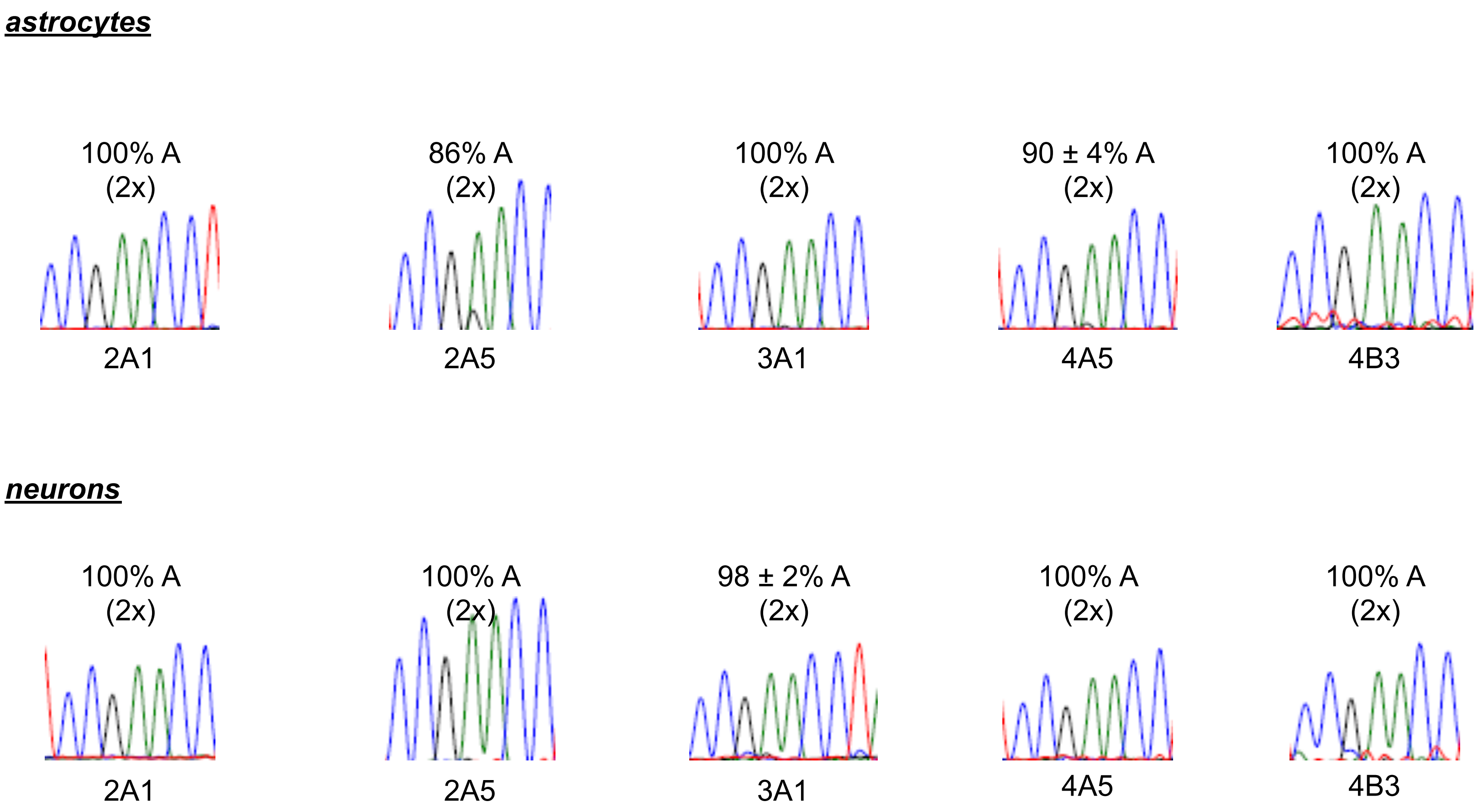
astrocytes
100% A
(2x)
2A1
86% A
(2x)
2A5
100% A
(2x)
3A1
90 ± 4% A
(2x)
4A5
100% A
(2x)
4B3
neurons
100% A
(2x)
2A1
100% A
(2x)
2A5
98 ± 2% A
(2x)
3A1
100% A
(2x)
4A5
100% A
(2x)
4B3

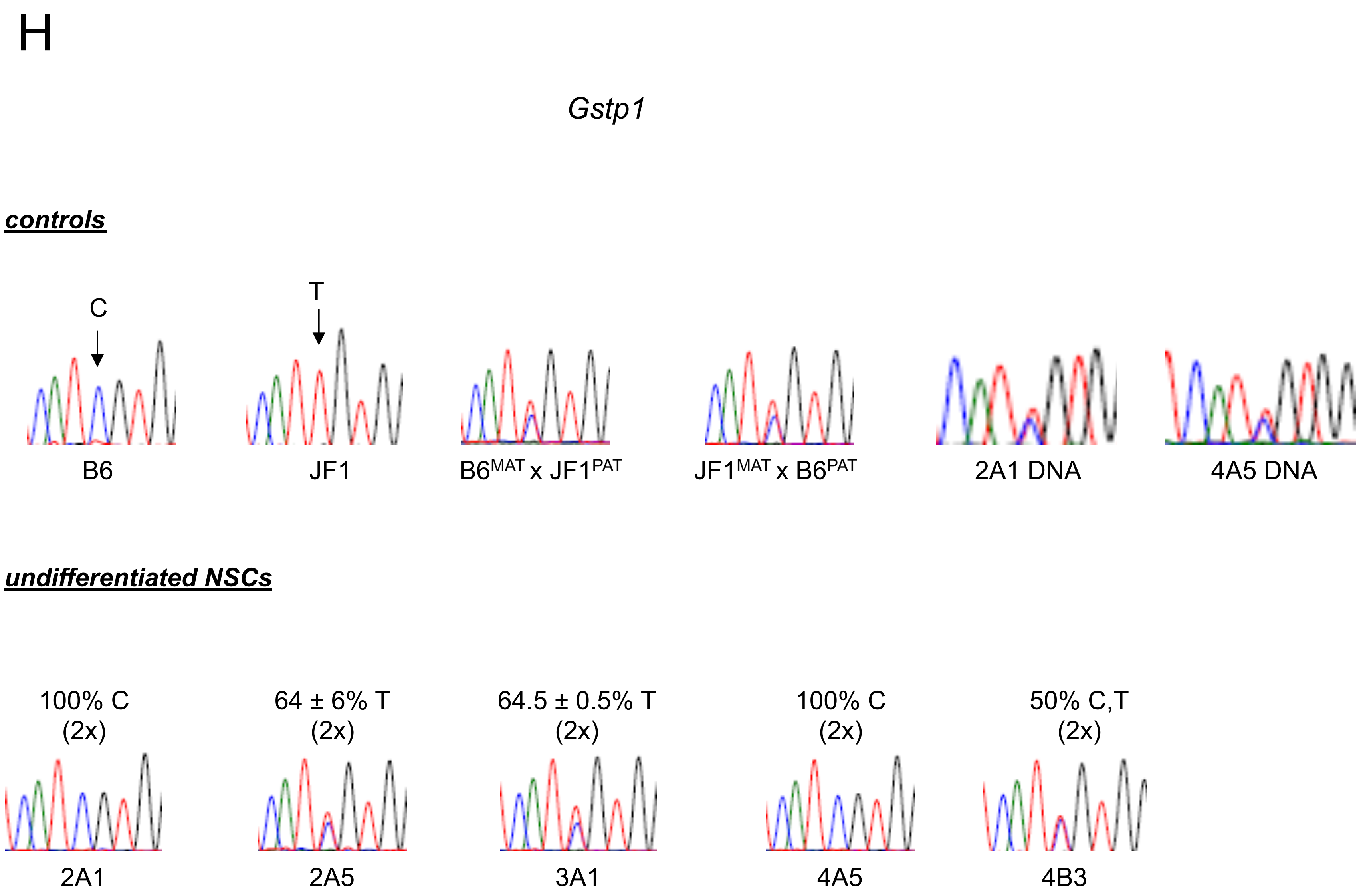
H
Gstp1
controls
C
T
B6
JF1
B6MAT x JF1PAT
JF1MAT x B6PAT
2A1 DNA
4A5 DNA
undifferentiated NSCs
100% C (2x)
64 ± 6% T (2x)
64.5 ± 0.5% T (2x)
100% C (2x)
50% C,T (2x)
2A1
2A5
3A1
4A5
4B3

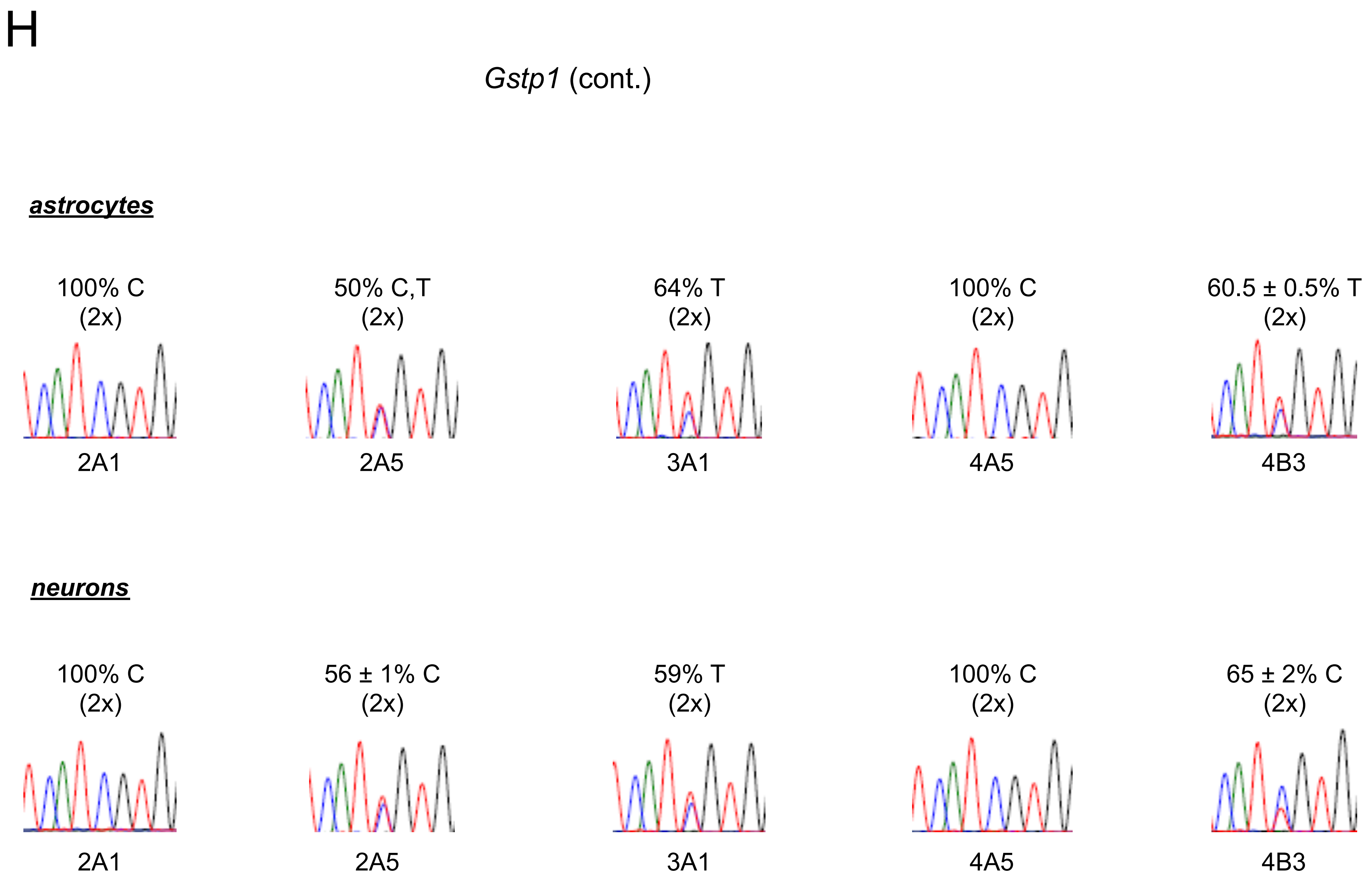
H
Gstp1 (cont.)
astrocytes
100% C (2x)
2A1
50% C,T (2x)
2A5
64% T (2x)
3A1
100% C (2x)
4A5
60.5 ± 0.5% T (2x)
4B3
neurons
100% C (2x)
2A1
56 ± 1% C (2x)
2A5
59% T (2x)
3A1
100% C (2x)
4A5
65 ± 2% C (2x)
4B3

I

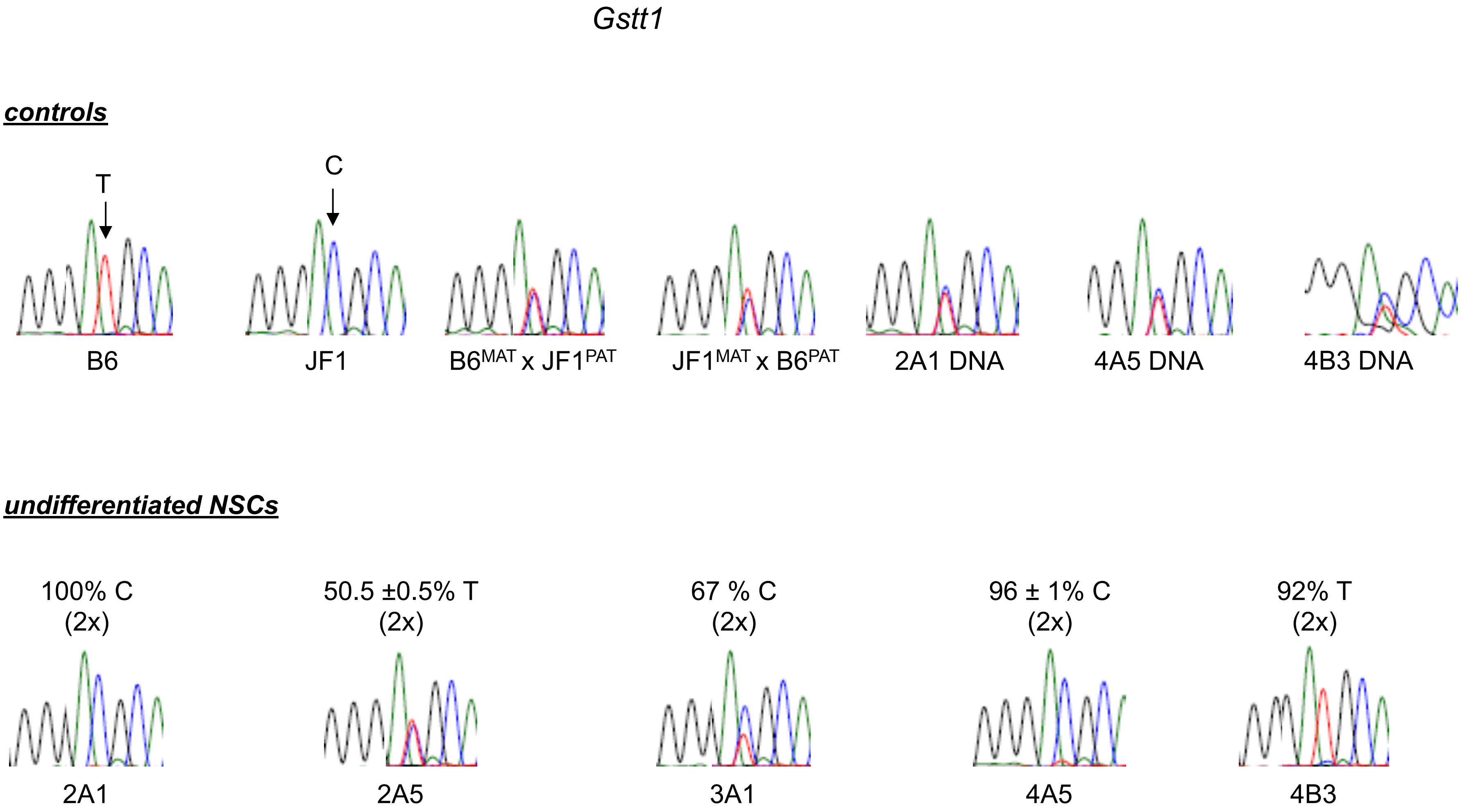
Gstt1
controls
T
C
B6
JF1
B6MAT x JF1PAT
JF1MAT x B6PAT
2A1 DNA
4A5 DNA
4B3 DNA
undifferentiated NSCs
100% C
(2x)
50.5 ±0.5% T
(2x)
67 % C
(2x)
96 ± 1% C
(2x)
92% T
(2x)
2A1
2A5
3A1
4A5
4B3

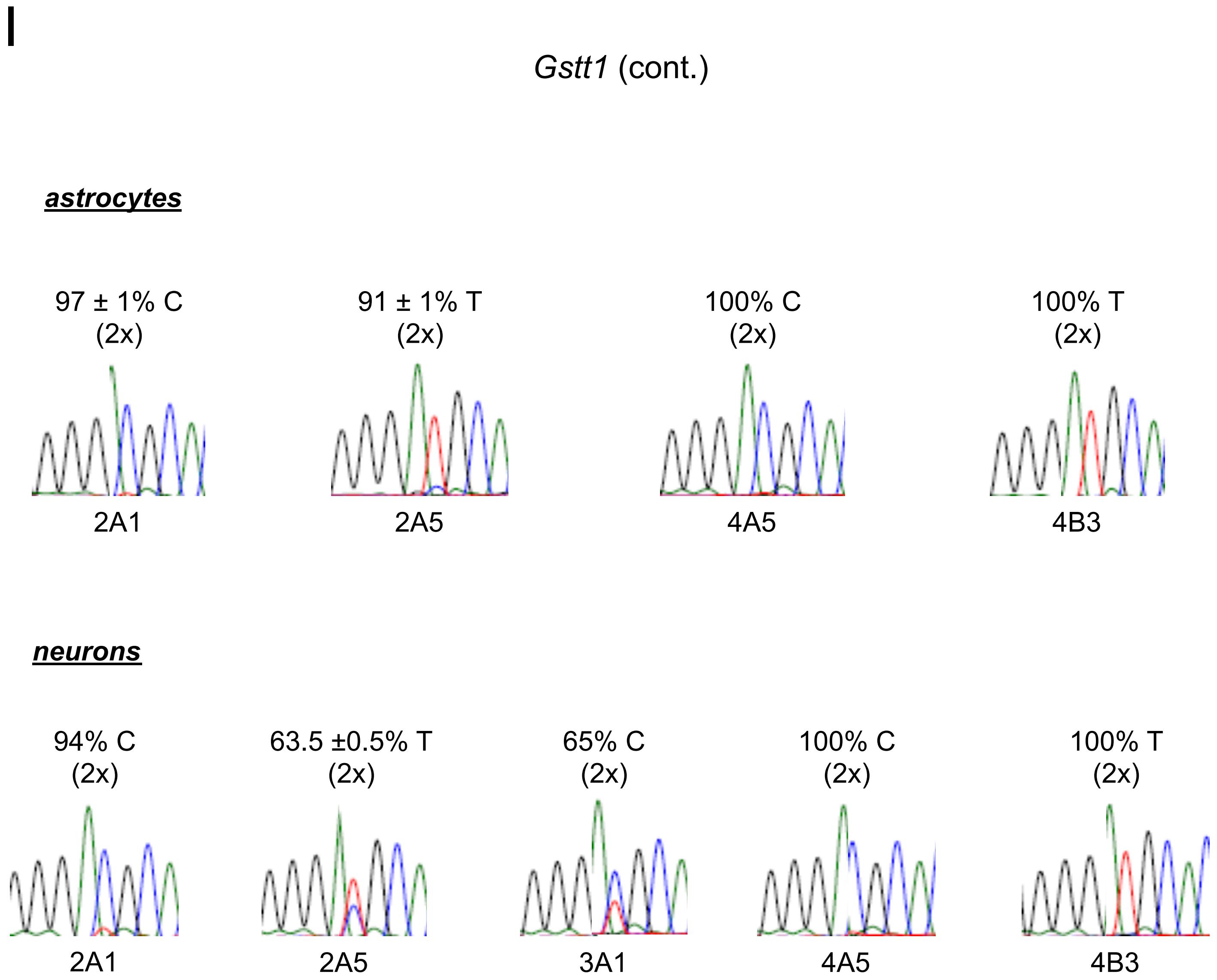
I
Gstt1 (cont.)
astrocytes
97 ± 1% C
(2x)
91 ± 1% T
(2x)
100% C
(2x)
100% T
(2x)
2A1
2A5
4A5
4B3
neurons
94% C
(2x)
63.5 ±0.5% T
(2x)
65% C
(2x)
100% C
(2x)
100% T
(2x)
2A1
2A5
3A1
4A5
4B3

J

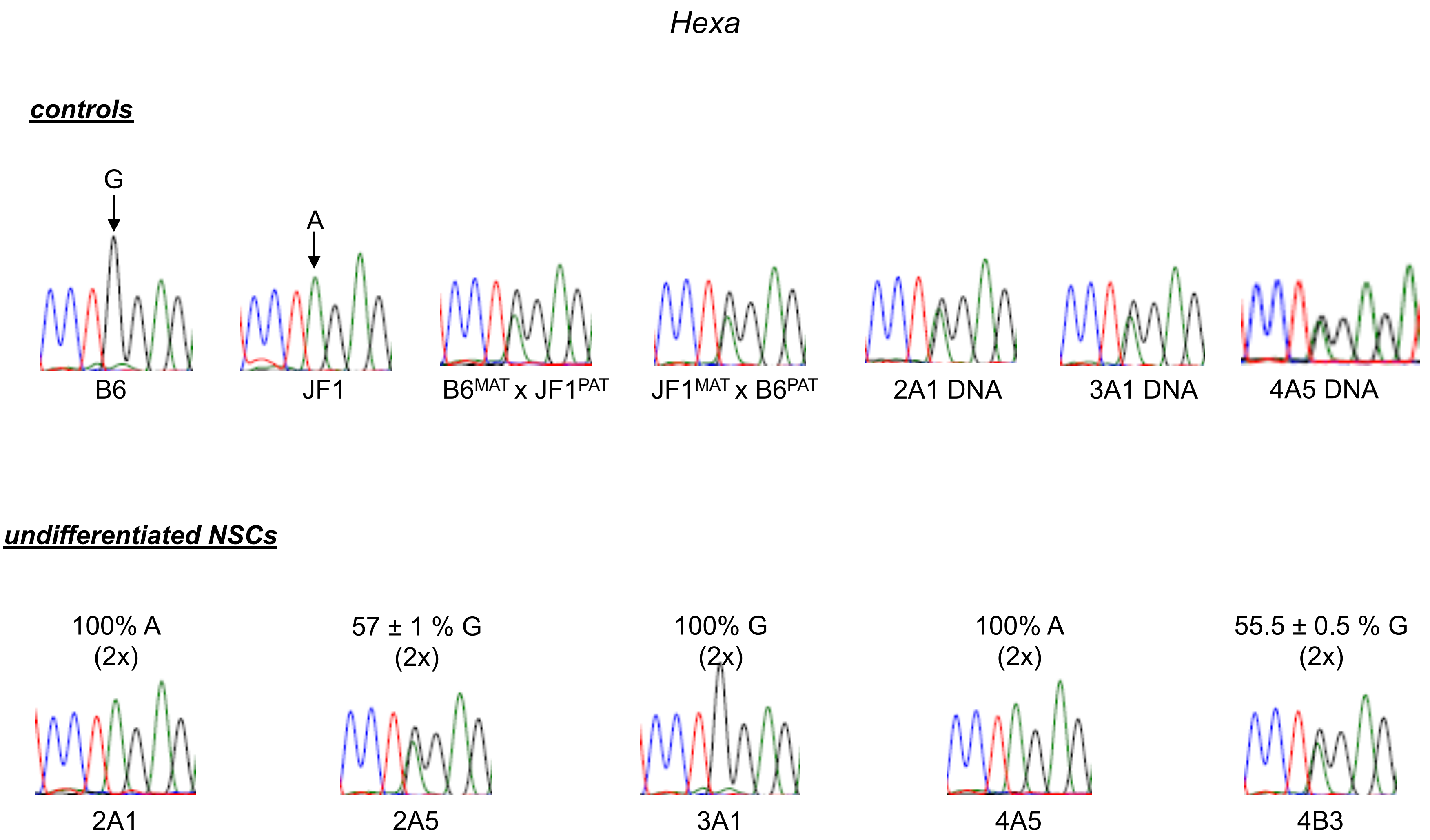
Hexa
controls
G
A
B6
JF1
B6MAT x JF1PAT
JF1MAT x B6PAT
2A1 DNA
3A1 DNA
4A5 DNA
undifferentiated NSCs
100% A
(2x)
57 ± 1 % G
(2x)
100% G
(2x)
100% A
(2x)
55.5 ± 0.5 % G
(2x)
2A1
2A5
3A1
4A5
4B3

J

Hexa (cont.)

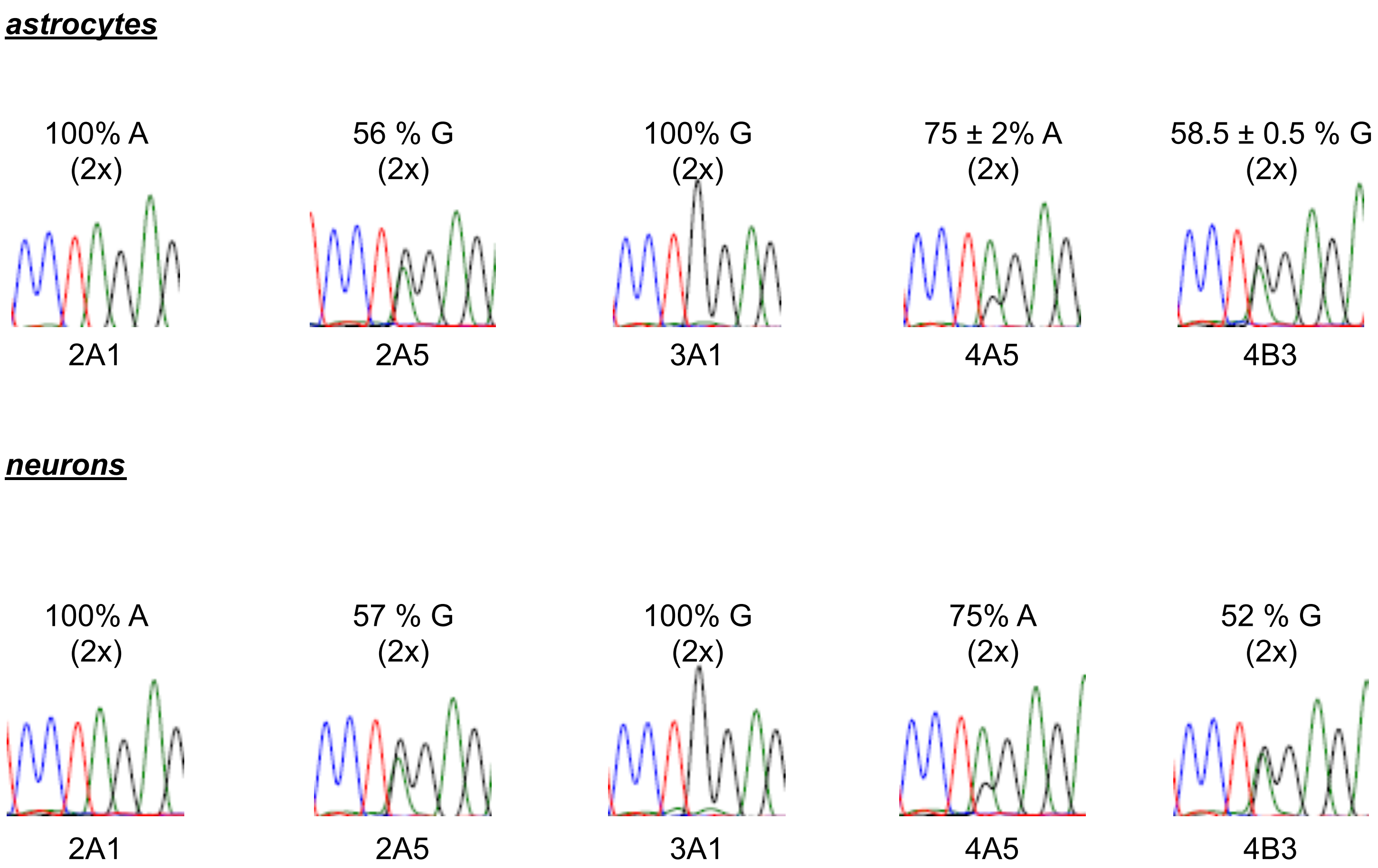

astrocytes
100% A (2x)
2A1
56 % G (2x)
2A5
100% G (2x)
3A1
75 ± 2% A (2x)
4A5
58.5 ± 0.5 % G (2x)
4B3
neurons
100% A (2x)
2A1
57 % G (2x)
2A5
100% G (2x)
3A1
75% A (2x)
4A5
52 % G (2x)
4B3

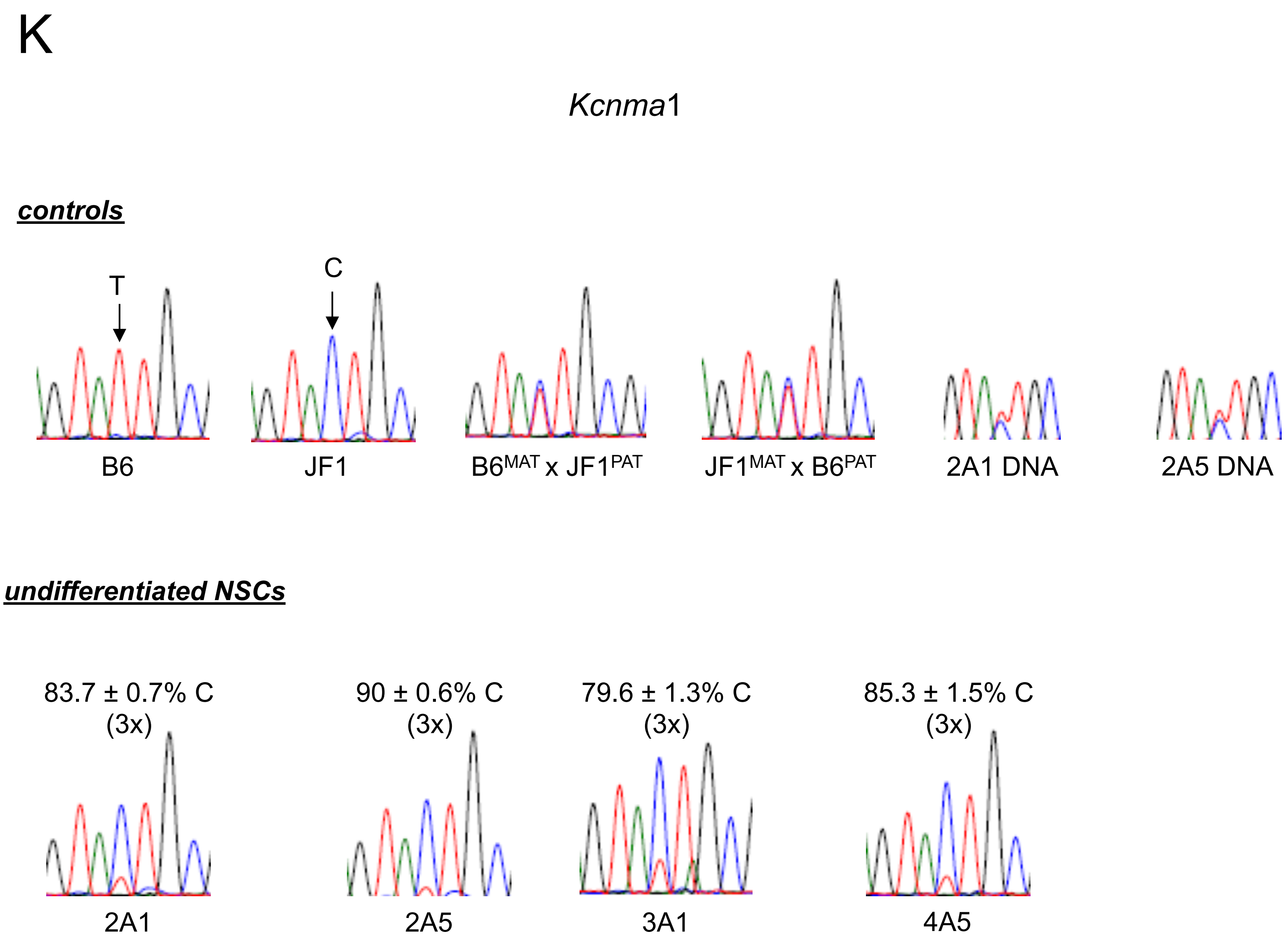
K
Kcnma1
controls
T
C
B6
JF1
B6MAT x JF1PAT
JF1MAT x B6PAT
2A1 DNA
2A5 DNA
undifferentiated NSCs
83.7 ± 0.7% C
(3x)
90 ± 0.6% C
(3x)
79.6 ± 1.3% C
(3x)
85.3 ± 1.5% C
(3x)
2A1
2A5
3A1
4A5

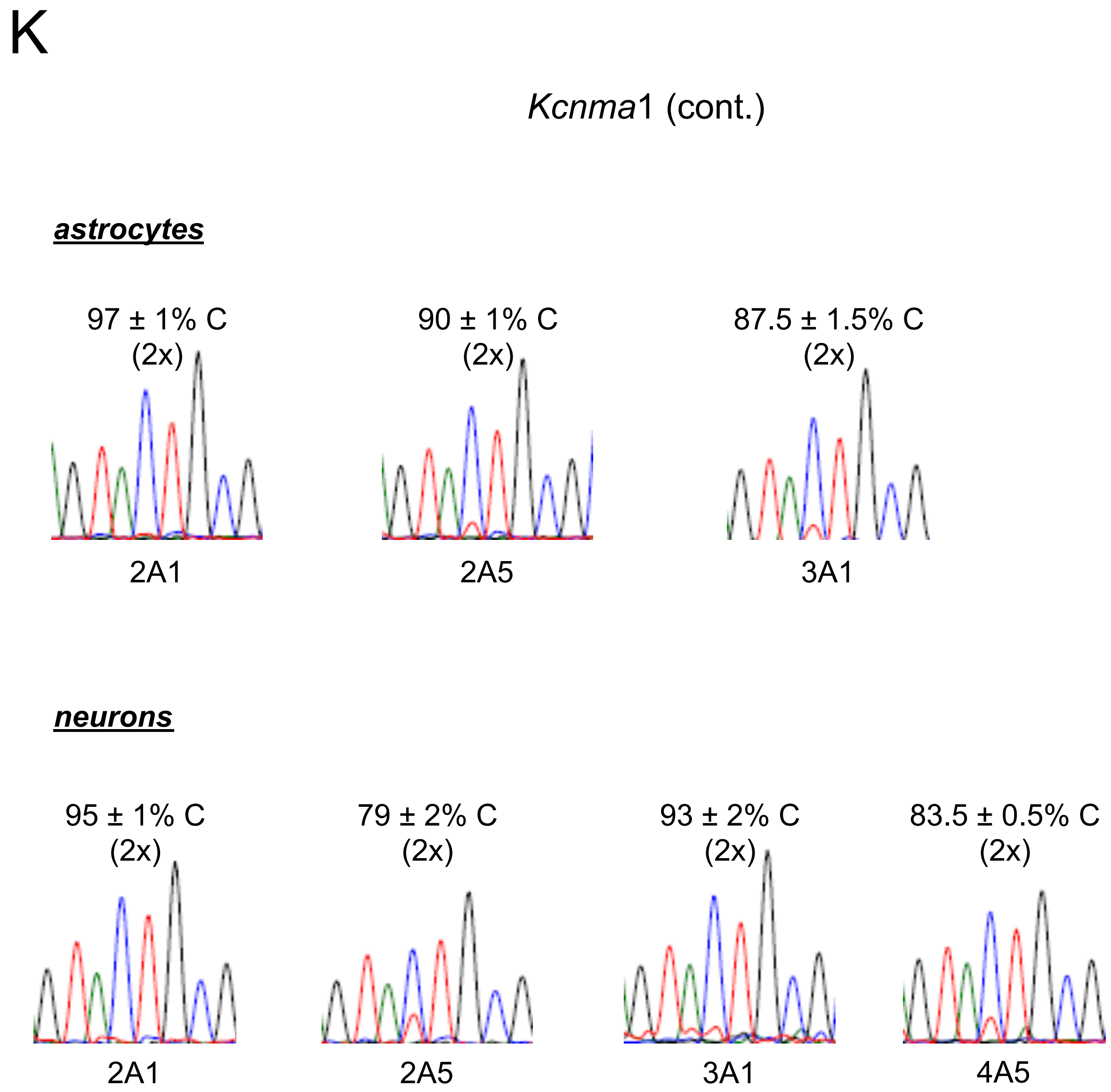
K
Kcnma1 (cont.)
astrocytes
97 ± 1% C
(2x)
2A1
90 ± 1% C
(2x)
2A5
87.5 ± 1.5% C
(2x)
3A1
neurons
95 ± 1% C
(2x)
2A1
79 ± 2% C
(2x)
2A5
93 ± 2% C
(2x)
3A1
83.5 ± 0.5% C
(2x)
4A5

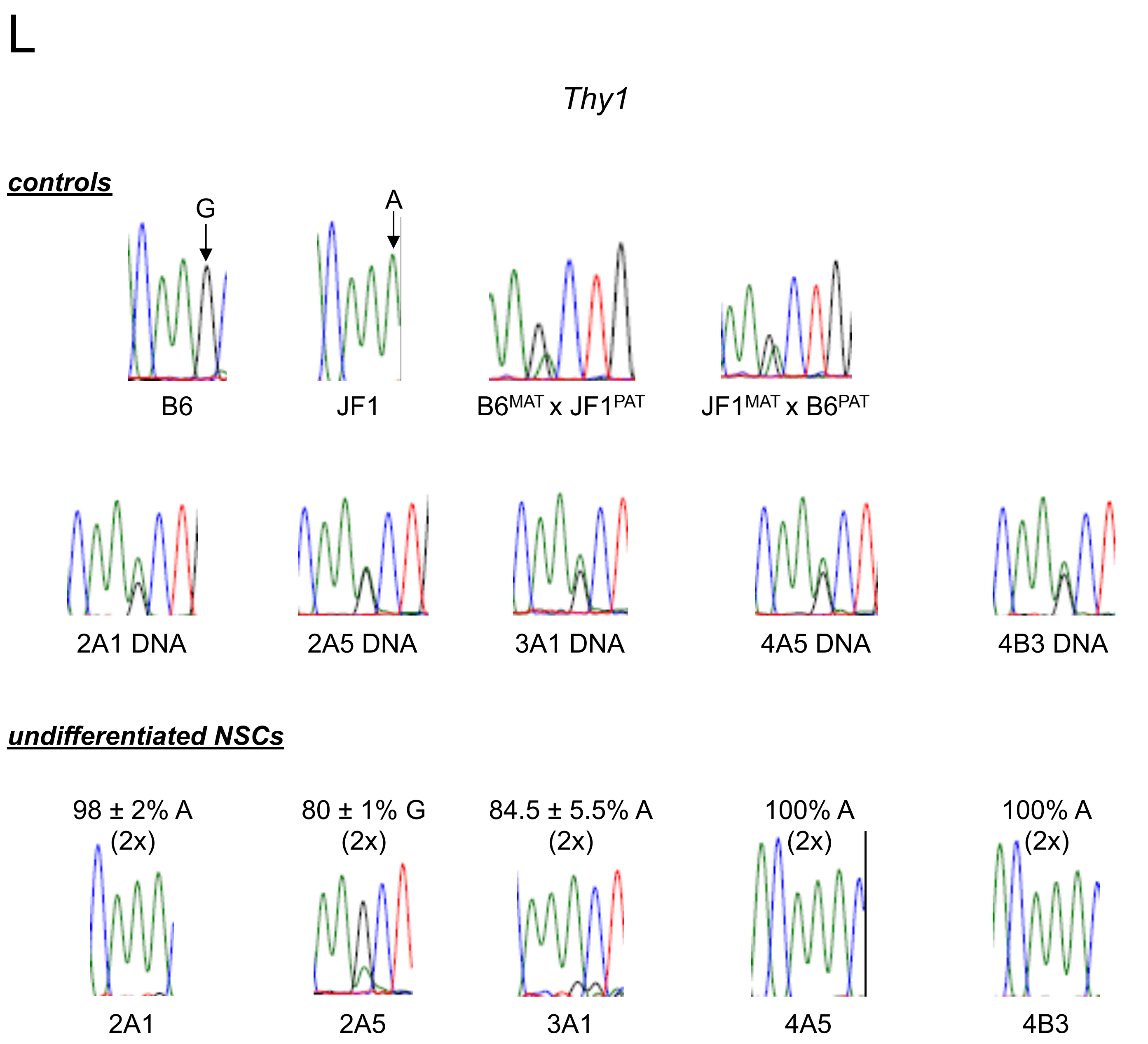
L
Thy1
controls
G
A
B6
JF1
B6MAT x JF1PAT
JF1MAT x B6PAT
2A1 DNA
2A5 DNA
3A1 DNA
4A5 DNA
4B3 DNA
undifferentiated NSCs
98 ± 2% A (2x)
80 ± 1% G (2x)
84.5 ± 5.5% A (2x)
100% A (2x)
100% A (2x)
2A1
2A5
3A1
4A5
4B3

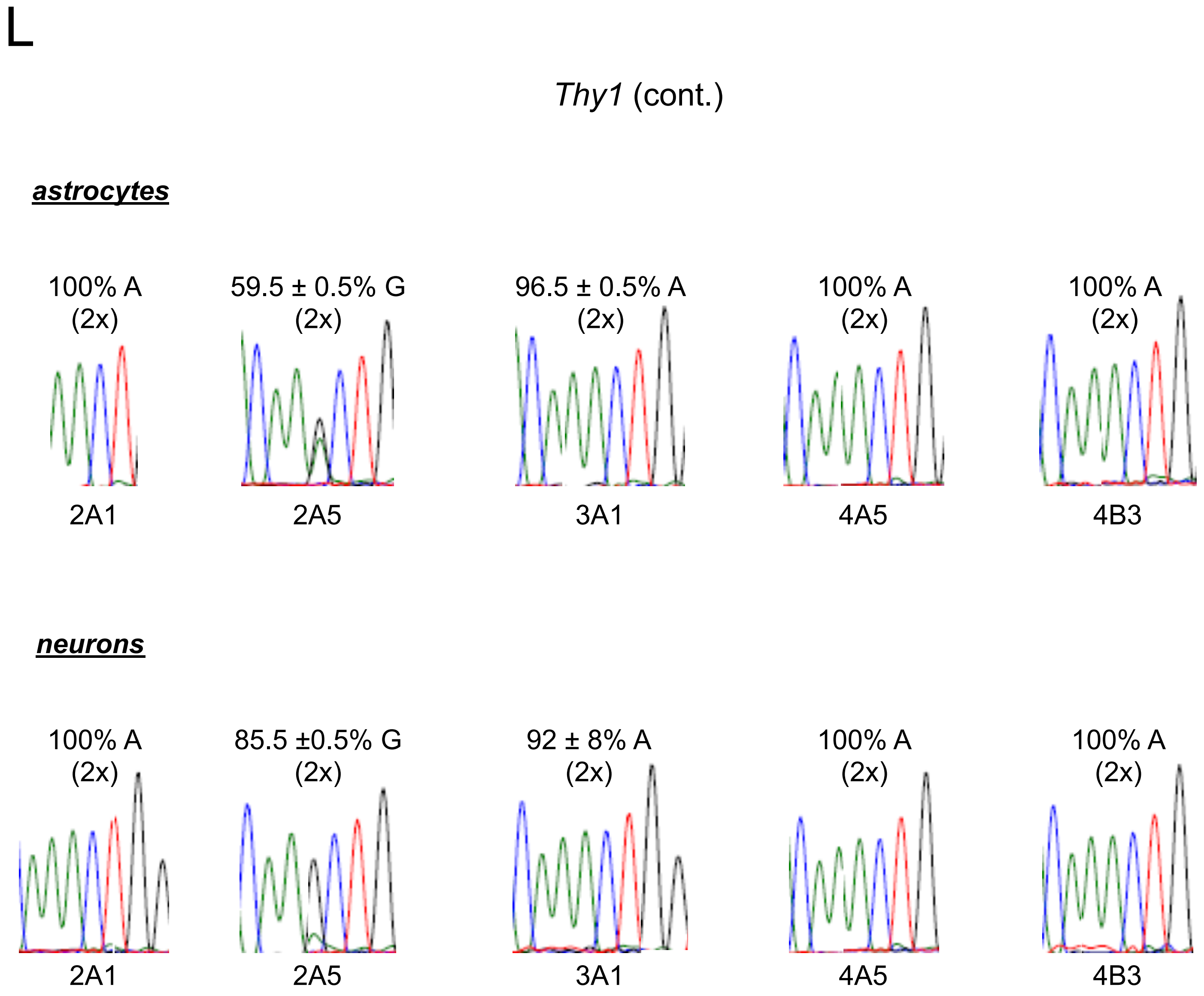
L
Thy1 (cont.)
astrocytes
100% A (2x)
59.5 ± 0.5% G (2x)
96.5 ± 0.5% A (2x)
100% A (2x)
100% A (2x)
2A1
2A5
3A1
4A5
4B3
neurons
100% A (2x)
85.5 ±0.5% G (2x)
92 ± 8% A (2x)
100% A (2x)
100% A (2x)
2A1
2A5
3A1
4A5
4B3
