# Supplementary material for: Transcriptome-Wide Survey of Mouse CNS-Derived Cells Reveals Monoallelic Expression within Novel Gene Families
Source: PLoS One. 2012 Feb 22;7(2):e31751. doi: 10.1371/journal.pone.0031751 (PMC3285176; doi:10.1371/journal.pone.0031751)
Supplement: Table S1 — Number of Refseq genes in each category of allele-specific expression by cell line. (DOC) [file pone.0031751.s005.doc]

Table S1: Number of Refseq genes in each category of allele-specific expression by cell line.

| Refseq gene category | Hybrid cell lines | | | |
| --- | --- | --- | --- | --- |
|  | 2A1 | 2A5 | 3A1 | 4A5a |
| Monoallelic expression |  |  |  |  |
| ChrX | 206 (0.70%) | 214 (0.24%) | 238 (1.08%) | 205 (2.25%) |
| Autosomes | 244 (1.33%) | 172 (3.49%) | 229 (2.02%) | 220 (3.76%) |
| Monoallelic trend |  |  |  |  |
| ChrX | 4 (<0.01%) | 4 (0.02%) | 3 (<0.01%) | 1 (<0.01%) |
| Autosomes | 668 (4.30%) | 503 (6.94%) | 549 (6.29%) | 457 (7.45%) |
| Monoallelic or trend in doubt |  |  |  |  |
| ChrX | 0 | 0 | 0 | 1 (9.41%) |
| Autosomes | 38 (22.05%) | 77 (32.45%) | 89 (29.74%) | 68(34.09%) |
| Biallelic expression |  |  |  |  |
| ChrX | 5 | 4 | 2 | 4 |
| Autosomes | 5968 | 6399 | 6244 | 5979 |
| Total # of evaluable genes | 7133 | 7373 | 7354 | 6935 |

Genes were considered to show 1) monoallelic expression (*P_B6_* or *P_JF1_* >= 0.85,

*p*-value <=0.05 exact binomial test, null hypothesis *P_B6_* = *P_JF1_*= 0.5); 2) a monoallelic trend (0.7 <= *P_B6_* or *P_JF1_* < 0.85, same *p*-value threshold); 3) biallelic expression (*P_B6_* or *P_JF1_* < 0.7), or 4) monoallelic expression or trend in doubt (*P_B6_* or *P_JF1_* >= 0.7, *p*-value>0.05). The FDR, shown in parentheses, was estimated with *p*-values from the exact binomial distribution.
