# Supplementary material for: Transcriptome-Wide Survey of Mouse CNS-Derived Cells Reveals Monoallelic Expression within Novel Gene Families
Source: PLoS One. 2012 Feb 22;7(2):e31751. doi: 10.1371/journal.pone.0031751 (PMC3285176; doi:10.1371/journal.pone.0031751)
Supplement: Table S2 — Enrichment of autosomal gene clusters showing monoallelic expression by use of the DAVID Bioinformatics Site. (DOC) [file pone.0031751.s006.doc]

Table S2: Enrichment of autosomal gene clusters showing monoallelic expression by use of the DAVID Bioinformatics Site.

|  |  | **Term** | **#**  **genes** | **Fold**  **enrichment** | **FDR (%)** | **Genes** |
| --- | --- | --- | --- | --- | --- | --- |
| **Genes with**  **monoallelic expr. in >=2 cell lines (n=170)** | **Protein**  Domain | | IPR004045:Glutathione S-transferase, N-terminal | | --- | | IPR004046:Glutathione S-transferase, C-terminal | | IPR017933:Glutathione S-transferase/chloride channel, C-terminal | | | 4 | | --- | | 4 | | 4 | | | 20.72 | | --- | | 19.98 | | 16.45 | | | 1.21 | | --- | | 1.34 | | 2.37 | | | GSTT1, GSTO1, GSTM5, GSTP1 | | --- | | GSTT1, GSTO1, GSTM5, GSTP1 | | GSTT1, GSTO1, GSTM5, GSTP1 | |
|  | **Kegg**  Pathway | \| mmu00480:Glutathione metabolism \| \| --- \| \| mmu00980:Metabolism of xenobiotics by cytochrome P450 \| \| mmu00982:Drug metabolism \| | \| 5 \| \| --- \| \| 5 \| \| 5 \| | \| 9.51 \| \| --- \| \| 7.49 \| \| 6.60 \| | \| 1.68 \| \| --- \| \| 4.00 \| \| 6.24 \| | \| GSTK1, GSTT1, GSTO1, GSTM5, GSTP1 \| \| --- \| \| GSTK1, GSTT1, GSTO1, GSTM5, GSTP1 \| \| GSTK1, GSTT1, GSTO1, GSTM5, GSTP1 \| |
| **Genes with monoallelic expr. in >=1 cell line (n=577)** | **Protein**  **Domain** | | IPR013164:Cadherin, N-terminal | | --- | | IPR002126:Cadherin | | IPR015493:Protocadherin beta | | IPR015492:Protocadherin gamma | | IPR017933:Glutathione S-transferase/chloride channel, C-terminal | | IPR001464:Annexin | | IPR018502:Annexin repeat | | IPR018252:Annexin repeat, conserved site | | IPR004045:Glutathione S-transferase, N-terminal | | IPR004046:Glutathione S-transferase, C-terminal | | | 15 | | --- | | 19 | | 9 | | 6 | | 6 | | 4 | | 4 | | 4 | | 5 | | 5 | | | 11.59 | | --- | | 7.05 | | 15.49 | | 16.23 | | 6.68 | | 12.62 | | 12.62 | | 12.62 | | 7.01 | | 6.76 | | | 2.90E-08 | | --- | | 2.48E-07 | | 7.87E-05 | | 0.03 | | 2.83 | | 5.09 | | 5.09 | | 5.09 | | 7.74 | | 8.80 | | | PCDHGA12, PCDHB6, PCDHB4, PCDHGB7, PCDHB2, PCDHGA7, PCDHB14, PCDHGA6,PCDHB13, PCDHB12, PCDHB11, PCDHB10, PCDHGA1, PCDHGB1, PCDHB16 | | --- | | PCDHGA12, 2610005L07RIK, PCDHB6, PCDHB4, PCDHGB7, PCDHB2, PCDHGA7, PCDHB14, PCDHB13, PCDHGA6, PCDHB12, PCDHB11, CDH4, PCDHB10, PCDHGA1, CDH6, PCDHGB1, PCDHB16, CDH10 | | PCDHB6, PCDHB16, PCDHB4, PCDHB14, PCDHB2, PCDHB13, PCDHB12, PCDHB11, PCDHB10 | | PCDHGA12, PCDHGB1, PCDHGB7, PCDHGA7, PCDHGA6, PCDHGA1 | | GSTM2, GSTT1, CLIC1, GSTO1, GSTM5, GSTP1 | | ANXA1, ANXA5, ANXA4, ANXA2 | | ANXA1, ANXA5, ANXA4, ANXA2 | | ANXA1, ANXA5, ANXA4, ANXA2 | | GSTM2, GSTT1, GSTO1, GSTM5, GSTP1 | | GSTM2, GSTT1, GSTO1, GSTM5, GSTP1 | |
|  | **Kegg**  **Pathway** | \| mmu04512:ECM-receptor interaction \| \| --- \| \| mmu04540:Gap junction \| \| mmu04510:Focal adhesion \| \| mmu00982:Drug metabolism \| | \| 9 \| \| --- \| \| 9 \| \| 14 \| \| 8 \| | \| 3.70 \| \| --- \| \| 3.57 \| \| 2.41 \| \| 3.64 \| | \| 3.119 \| \| --- \| \| 3.88 \| \| 5.57 \| \| 6.71 \| | \| VWF, SDC1, COL4A1, LAMB1-1, ITGA7, ITGA4, COL5A3, COL5A2, HMMR \| \| --- \| \| GM6682, ADRB1, GJA1, GUCY1A3, PDGFC, TUBA1A, TUBB3, TUBA1C, PRKCB, TUBB4 \| \| CAV2, VAV3, COL4A1, ERBB2, ITGA4, COL5A3, COL5A2, MYL9, PRKCB, VWF, ITGA7, LAMB1-1, PDGFC, PARVB \| \| GSTM2, FMO1, GSTK1, AOX1, GSTT1, GSTO1, GSTM5, GSTP1 \| |
| **Genes with trend to monoallelic expr. in >=3 cell lines (n=144)** | **Protein**  **Domain** | | NA | | --- | | | NA | | --- | | | NA | | --- | | | NA | | --- | | | NA | | --- | |
|  | **Kegg**  **Pathway** | \| NA \| \| --- \| | \| NA \| \| --- \| | \| NA \| \| --- \| | \| NA \| \| --- \| | \| NA \| \| --- \| |
| **Genes with**  **monoallelic expr. or monoallelic trend in in >=2 cell lines**  **(n=443)** | **Protein**  **Domain** | | IPR015493:Protocadherin beta | | --- | | IPR013164:Cadherin, N-terminal | | IPR013781:Glycoside hydrolase, subgroup, catalytic core | | IPR002347:Glucose/ribitol dehydrogenase | | IPR002198:Short-chain dehydrogenase/reductase SDR | | | 7 | | --- | | 7 | | 6 | | 7 | | 7 | | | 14.91 | | --- | | 6.70 | | 8.27 | | 5.86 | | 5.13 | | | 0.01 | | --- | | 0.87 | | 1.07 | | 1.77 | | 3.52 | | | PCDHB7, PCDHB8, PCDHB6, PCDHB14, PCDHB2, PCDHB11, PCDHB10 | | --- | | PCDHB7, PCDHB8, PCDHB6, PCDHB14, PCDHB2, PCDHB11, PCDHB10 | | GLB1L, GALC, FUCA2, MANBA, GLB1, IDUA | | DHRS1, DHRS4, DHRS13, DECR2, H2-KE6, HSD17B4, DHRS7 | | DHRS1, DHRS4, DHRS13, DECR2, H2-KE6, HSD17B4, DHRS7 | |
|  | **Kegg**  **Pathway** | \| NA \| \| --- \| | \| NA \| \| --- \| | \| NA \| \| --- \| | \| NA \| \| --- \| | \| NA \| \| --- \| |

**^*^**We used the DAVID Bioinformatics Website version 6.7 (david.abcc.ncifcrf.gov) to detect enrichment of genes showing monoallelic expression (or a trend) vs. total Mus musculus genes. Only genes with FDR < 10% are included.
