# Supplementary material for: Transcriptome-Wide Survey of Mouse CNS-Derived Cells Reveals Monoallelic Expression within Novel Gene Families
Source: PLoS One. 2012 Feb 22;7(2):e31751. doi: 10.1371/journal.pone.0031751 (PMC3285176; doi:10.1371/journal.pone.0031751)
Supplement: Table S3 — Allele-specific expression of selected genes in NSC lines before and after differentiation to astrocytes or neurons. (DOC) [file pone.0031751.s007.doc]

| Table S3. Allele-specific expression of selected genes in NSC lines before and after differentiation | | | | | | |
| --- | --- | --- | --- | --- | --- | --- |
| to astrocytes or neurons. | |  |  |  |  |  |
| Gene | Cell type |  | Neural stem cell line | |  |  |
|  |  | 2A1 | 2A5 | 3A1 | 4A5 | 4B3 |
| *Anxa1* | *undiff* | 91% B†† | 98% B†† | biallelic† | biallelic | 97% B |
|  | *astrocytes* | biallelic | biallelic | biallelic | biallelic | biallelic |
|  | *neurons** | 100% B | biallelic | *n.d.* | biallelic | 100% B |
| *Anxa2* | *undiff* | biallelic† | biallelic† | 97% B†† | biallelic | biallelic |
|  | *astrocytes*** | biallelic | biallelic | 90% B | *n.d.* | biallelic |
|  | *Neurons*** | biallelic | biallelic | 97% B | biallelic | biallelic |
| *Chl1* | *undiff* | biallelic† | biallelic† | 90% J†† | biallelic | biallelic |
|  | *astrocytes*** | biallelic | biallelic | 86% J | biallelic | biallelic |
|  | *neurons*** | biallelic | biallelic | 97% J | biallelic | biallelic |
| *Gabrg1* | *undiff* | 97% B†† | biallelic† | *n.d.* | 100% B | biallelic |
|  | *astrocytes*** | 100% B | biallelic | *n.d.* | 100% B | 81% B |
|  | *neurons** | 100% B | biallelic | *n.d.* | 100% B | 100% B |
| *Gm2a* | *undiff* | 90% B†† | biallelic† | 100% B†† | 99% B | biallelic |
|  | *astrocytes*** | 99% B | biallelic | 96% B | biallelic | *n.d* |
|  | *neurons* | biallelic | biallelic | 100% B | biallelic | *n.d* |
| *Hexa* | *undiff* | 100% J†† | biallelic† | 100% B†† | 100% J | biallelic |
|  | *astrocytes*** | 100% J | biallelic | 100% B | biallelic | biallelic |
|  | *neurons*** | 100% J | biallelic | 100% B | biallelic | biallelic |
| *Kcnma1* | *undiff* | 84% J†† | 90% J† | 80% J† | 85% J | *n.d.* |
|  | *astrocytes* | 97% J | 90% J | 88% J | *n.d.* | *n.d.* |
|  | *neurons* | 95% J | biallelic | 93% J | 84% J | *n.d.* |
| *Thy1* | *undiff* | 98% J†† | 80% B†† | 85% J | 100% J | 100% J |
|  | *astrocytes*** | 100% J | biallelic | 97% J | 100% J | 100% J |
|  | *neurons** | 100% J | 86% B | 92% J | 100% J | 100% J |
| Numerical values (rounded to the nearest integer) are shown for RT-PCR samples with at least a 0.8 | | | | | | |
| preference for the B6 (B) or JF1 (J) allele. Other values are listed as biallelic (see Figure S4 for | | | | | | |
| representative data and technical replicates.) | | | |  |  |  |
| Asterisks denote concordance with allele-specific expression in undifferentiated NSCs: | | | | | | |
| **p*-value <0.05; ***p*-value <0.001. | | |  |  |  |  |
| Daggers indicate results for samples evaluated by Illumina sequencing: † Biallelic expression | | | | | | |
| at PB6 or PJF1 < 0.85, †† Monoallelic expression at PB6 or PJF1 >= 0.85. | | | | | |  |
| *n.d.,* not detected. | |  |  |  |  |  |
