# Supplementary material for: Transcriptome-Wide Survey of Mouse CNS-Derived Cells Reveals Monoallelic Expression within Novel Gene Families
Source: PLoS One. 2012 Feb 22;7(2):e31751. doi: 10.1371/journal.pone.0031751 (PMC3285176; doi:10.1371/journal.pone.0031751)
Supplement: Table S4 — List of primers. (DOC) [file pone.0031751.s008.doc]

| Table S4. List of primers. | | |  | |  | |  | |  | |  | |  | |  | |  | |
| --- | --- | --- | --- | --- | --- | --- | --- | --- | --- | --- | --- | --- | --- | --- | --- | --- | --- | --- |
|  |  |  | |  | |  | |  | |  | |  | |  | |  | |  |
| Gene | NCBI Refseq ID | SNP (B6/JF1) | | SNP exon | | Ref. position | | Primer No. | | Substrate | | Primer sequence | | product size | |  | |  |
|  |  |  | |  | |  | |  | |  | |  | | (bp) | |  | |  |
|  |  |  | |  | |  | |  | |  | |  | |  | |  | |  |
| Anxa1 | NM_010730 | G/A | | 2 | | 104 | | 291 | | RNA | | TTGCTAGGTGTGGCTTCAGTT | | 282 | |  | |  |
|  |  |  | |  | |  | | 292 | | RNA | | CAGCAACATCCGAGGATACAT | |  | |  | |  |
|  |  |  | |  | |  | | 307 | | DNA | | GCGTAGTGTTTGTGGATGTTG | | 283 | |  | |  |
|  |  |  | |  | |  | | 308 | | DNA | | GACCCAGGACCACCTTTGTAT | |  | |  | |  |
| Anxa2 | NM_007585 | T/C | | 3 | | 176 | | 323 | | RNA | | CTACTGTCCACGAAATCCTGTG | | 286 | |  | |  |
|  |  |  | |  | |  | | 324 | | RNA | | GTCTCCAGGTGGCCAGATAAG | |  | |  | |  |
|  |  |  | |  | |  | | 397 | | DNA | | AGAGGGTGTGCCTGGTTTAAT | | 221 | |  | |  |
|  |  |  | |  | |  | | 398 | | DNA | | TTGTGACTCAGCTGCAATACG | |  | |  | |  |
| Chl1 | NM_00769 | C/T | | 5 | | 796 | | 261 | | RNA | | CATTCAAGATCCCAAATGAGG | | 231 | |  | |  |
|  |  |  | |  | |  | | 262 | | RNA | | TCATCCAATAGATGTGCAAAGG | |  | |  | |  |
|  |  |  | |  | |  | | 275 | | DNA | | ATGAGGGCCACATCTCTCAC | | 291 | |  | |  |
|  |  |  | |  | |  | | 276 | | DNA | | GAAGCTGACAGAGTGGTTATGC | |  | |  | |  |
| Gabrg1 | NM_01025 | T/C | | 8 | | 1355 | | 229 | | RNA | | AGTGGTTCTTTCCTGGGTGTC | | 214 | |  | |  |
|  |  |  | |  | |  | | 230 | | RNA | | ATGCAAGGTTCCGTATTCCAT | |  | |  | |  |
|  |  |  | |  | |  | | 279 | | DNA | | CCCCCTTTCTTCACAGGTATC | | 165 | |  | |  |
|  |  |  | |  | |  | | 230 | | DNA | | ATGCAAGGTTCCGTATTCCAT | |  | |  | |  |
| Gm2a | NM_010299 | C/T | | 3 | | 371 | | 317 | | RNA | | CCTGGAGATGTAGTCGTCAGC | | 271 | |  | |  |
|  |  |  | |  | |  | | 318 | | RNA | | TGCTCGTGGGTAGTGAGTAGG | | | |  | |  |
|  |  |  | |  | |  | | 319 | | DNA | | CTTATTGCAGCGGGTTATGAG | | 292 | |  | |  |
|  |  |  | |  | |  | | 320 | | DNA | | ACCTTGACACCCCAGGTACTC | |  | |  | |  |
| Gstk1 | NM_029555 | C/T | | 8 | | 752 | | 267 | | RNA | | AGCTCATTGAGAACACGGATG | | 278 | |  | |  |
|  |  |  | |  | |  | | 268 | | RNA | | CAGAAGTAAAGGCAGGCACAG | |  | |  | |  |
|  |  |  | |  | |  | | 280 | | DNA | | TGGGACTCCTTAGGACCATCT | | 224 | |  | |  |
|  |  |  | |  | |  | | 268 | | DNA | | CAGAAGTAAAGGCAGGCACAG | |  | |  | |  |
| Gstm5 | NM_010360 | T/C | | 7 | | 604 | | 265 | | RNA | | AGCTACCTGCACAGCTGAAAC | | 285 | |  | |  |
|  |  |  | |  | |  | | 266 | | RNA | | CTCTGGCTCAGCATAAGCACT | |  | |  | |  |
|  |  |  | |  | |  | | 281 | | DNA | | TGCCTGTCTATCTCTGCAGGT | | 292 | |  | |  |
|  |  |  | |  | |  | | 282 | | DNA | | CATGCTGTCTGACTCCACTGA | |  | |  | |  |
| Gsto1 | NM_010362 | A/G | | 4 | | 521 | | 269 | | RNA | | CTTGGTCACCGAATCTGTCAT | | 300 | |  | |  |
|  |  |  | |  | |  | | 270 | | RNA | | TGAAACCAAGGCCAAGTAAGA | |  | |  | |  |
|  |  |  | |  | |  | | 283 | | DNA | | CAAGCACTCTGGAGGGATTCT | | 265 | |  | |  |
|  |  |  | |  | |  | | 284 | | DNA | | AAAGGCACAAAGAAATGCAGA | |  | |  | |  |
| Gstp1 | NM_013541 | C/T | | 6 | | 481 | | 271 | | RNA | | CCCAGATGGATATGGTGAATG | | 285 | |  | |  |
|  |  |  | |  | |  | | 272 | | RNA | | GAGCCACATAGGCAGAGAGC | |  | |  | |  |
|  |  |  | |  | |  | | 285 | | DNA | | TATGGTAGGGAGGGAGAGCAT | | 467 | |  | |  |
|  |  |  | |  | |  | | 286 | | DNA | | CCCTTAGCTCTGCTCCACTTT | |  | |  | |  |
| Gstt1 | NM_008185 | T/C | | 5 | | 893 | | 311 | | RNA | | CCCTGCTGACCTCATCATAAA | | 225 | |  | |  |
|  |  |  | |  | |  | | 312 | | RNA | | TGAGGTCATGTGTGGCTATCA | |  | |  | |  |
|  |  |  | |  | |  | | 311 | | DNA | | CCCTGCTGACCTCATCATAAA | | 225 | |  | |  |
|  |  |  | |  | |  | | 312 | | DNA | | TGAGGTCATGTGTGGCTATCA | |  | |  | |  |
| Hexa | NM_010421 | G/A | | 8 | | 975 | | 315 | | RNA | | CCCCTGGGTTATTAACACCTT | | 266 | |  | |  |
|  |  |  | |  | |  | | 316 | | RNA | | CAGCGTCTGGATGTAGAAGGA | |  | |  | |  |
|  |  |  | |  | |  | | 321 | | DNA | | TTAGGTGCCCCTGGGTTATTA | | 272 | |  | |  |
|  |  |  | |  | |  | | 322 | | DNA | | AGAGGTGAGCCCGTAAGTAGC | |  | |  | |  |
| Kcnma1 | NM_010610 | T/C | | 12 | | 1562 | | 213 | | RNA | | CTGTTCAAACGGCATTTCACT | | 287 | |  | |  |
|  |  |  | |  | |  | | 214 | | RNA | | CATATTGCGTCATCACCCTCT | |  | |  | |  |
|  |  |  | |  | |  | | 309 | | DNA | | GAGAGACTGCAGTTGAGTCCAA | | 228 | |  | |  |
|  |  |  | |  | |  | | 310 | | DNA | | GGAGACCCAAAGAAAAGGTTG | |  | |  | |  |
| Thy1 | NM_009382 | G/A | | 4 | | 499 | | 297 | | RNA | | TCTCCAACCAGCCCTATATCA | | 257 | |  | |  |
|  |  |  | |  | |  | | 298 | | RNA | | CCCAACCAGTCACAGAGAAAT | |  | |  | |  |
|  |  |  | |  | |  | | 313 | | DNA | | CTTGTGGTGTGGAGGTTCTGT | | 268 | |  | |  |
|  |  |  | |  | |  | | 314 | | DNA | | CCCAACCAGTCACAGAGAAAT | |  | |  | |  |
|  |  |  | |  | |  | |  | |  | |  | |  | |  | |  |
| Reverse transcription and PCR were carried out as previously described (Wang et al., 2007), with 1.5 mM MgCl_2_, and an | | | | | | | | | | | | | | | |  | |  |
| annealing temperature of 60° C. For each set, the forward and reverse primers are in the first and second rows respectively. | | | | | | | | | | | | | | | |  | |  |
| The reference position is the SNP location relative to the start site of transcription of the NCBI Refseq IDs listed. | | | | | | | | | | | | | | | |  | |  |
| Primers were designed with the aid of Primer3 (http://frodo.wi.mit.edu/primer3). | | | | | | | | | | | |  | |  | |  | |  |
