# Supplementary material for: Transcriptome-Wide Survey of Mouse CNS-Derived Cells Reveals Monoallelic Expression within Novel Gene Families
Source: PLoS One. 2012 Feb 22;7(2):e31751. doi: 10.1371/journal.pone.0031751 (PMC3285176; doi:10.1371/journal.pone.0031751)
Supplement: Table S5 — Genes with random (i.e., haplotype-independent) monoallelic expression detected by RNA-seq. (DOC) [file pone.0031751.s009.doc]

Table S5. Genes with random (i.e., haplotype-independent) monoallelic expression detected by RNA-seq.

| *1110021L09Rik* |
| --- |
| *4922501L14Rik* |
| *Cdkn2b*  *Ctsz* |
| *Dhrs11* |
| *Dner* |
| *Dusp10* |
| *Fkbp11* |
| *Gas6* |
| *Gja1* |
| *Gstt1* |
| *Hexa* |
| *N6amt2* |
| *Ppic* |
| *Slc6a1* |
| *Thy1* |
| *Tmem121* |
| *Tuba1c* |

The genes listed show expression from either the B6 or the JF1 allele in at least 2 of 3 evaluable cell lines. Details for each gene are in Dataset S1.
