# Supplementary material for: Transcriptome-Wide Survey of Mouse CNS-Derived Cells Reveals Monoallelic Expression within Novel Gene Families
Source: PLoS One. 2012 Feb 22;7(2):e31751. doi: 10.1371/journal.pone.0031751 (PMC3285176; doi:10.1371/journal.pone.0031751)
Supplement: Methods S1 — Details of Illumina sequencing, alignment and data analysis. (DOC) [file pone.0031751.s010.doc]

Methods S1. Details of Illumina sequencing, alignment and data analysis.

**1. Generation of a C57BL/6 vs. JF1 cSNP library**

The JF1 cDNA library was generated by RNA-seq of polyA+ RNA purified from forebrain of a JF1 mouse. To perform RNA-seq on JF1 and NSC polyA+ RNAs, each RNA (100 ng) from JF1 forebrain or the NSC lines (~ 2 X 10^6^ cells/sample) was fragmented by heating at 95° for 6 minutes, then converted to double-stranded cDNA by standard methods. The cDNA was sonicated, and the ends enzymatically repaired with the End-It DNA End-Repair kit (Epicentre, Cat# ER0720). Following addition of an A base to the cDNA 3’ terminus, an adaptor oligonucleotide mix supplied by Illumina was ligated to the cDNA. PCR (15 cycles) was carried out by use of the high fidelity Phusion DNA polymerase (NEB Cat# F-531). Following purification, the amplified DNA was sized on a 2% agarose gel, and a 250-300 bp band was excised and purified. High throughput DNA sequencing was carried out by use of an Illumina Genome Analyzer (GA II).

Refseq sequences for Mus musculus C57BL/6 (B6) (NCBI Build 37 Mm9) were constructed with genome assembly data downloaded from the UCSC genome browser ([http://genome.ucsc.edu](http://genome.ucsc.edu/)). The Burrows-Wheeler Aligner (BWA) [1] was used to map the short reads to the B6 transciptome. Samtools [2] was used subsequently to identifiy cSNPs in mapped JF1 reads. The BWA defines map quality score as$mapQ=-10*\log_{10}P_{e}$, where $Pe$is the probability of mapping a read to the wrong place in reference sequences. Four runs of Illumina sequencing were done for JF1, including 1 single end run with 40 bp reads, 1 single end run with 80 bp reads and two paired end runs with 40 bp reads, generating ~132.77 million short reads with 5.63 billion bp of sequence data. We used the threshold of mapQ>=1 to exclude ambiguous reads where a short read is mapped to the reference sequences at two or more places. We further required the mapped reads to be ungapped to exclude short reads that cannot be mapped to reference sequences without insertion or deletion. About 34.40 million (25.91%) ungapped reliable reads with map quality score >=1 were mapped to B6 Refseq sequences, covering 38.03 million positions with ~1.48 billion bp of data (shown in SI methods Table 1 and Figure S1 A). The mean depth of coverage was 38.50 (sd=183.86) and the median depth of coverage was 13 (IQR=31).

From ~1.48 billion bp of high quality sequence data for JF1, 20,936 Refseq genes were sequenced. We identified mutations in JF1 at 298,151 positions using the SNP calling algorithm in Samtools, and 140,345 positions remained with SNP quality score >=20 and depth of coverage between [3, 2000]. The SNP quality score >=20 means the probability of a JF1 SNP position consensus identical to the B6 reference is less than 0.01. Furthermore, we required that the consensus of JF1 reads at the SNP location be greater than 90%, and obtained 118,579 SNPs for JF1. The median coverage for the SNPs was 15, and the 25% and 75% quantiles were 7 and 36, respectively. The 118,579 SNPs resided on 12,416 Refseq genes, among which 1947 genes (15.68%) had 1 SNP, 3907 genes (31.47%) had 2-5 SNPs, 2636 genes (21.23%) had 6-10 SNPs and 3926 genes (31.62%) had more than 10 SNPs (Figure S1 C).

**2. Evaluation of expression levels and identification of SNPs for hybrid cell lines**

Illumina sequencing was performed for 4 hybrid cell lines, 2A1, 2A5, 3A1 and 4A5a, each for 1 to 3 single or paired end runs with 40 or 80 bp reads. Sequencing data was mapped to B6 Refseq genes as described above. We obtained approximately 62.80, 58.90, 40.59 and 36.87 million short reads for the 4 hybrid cell lines, and the number of bp covered ranged from 2.89 billion to 4.98 billion. Using the same criteria with map quality score mapQ>=1 without gaps, we obtained approximately 17,70 (28.18%), 12.44 (21.12%), 11.28 (27.79%) and 11.11(30.13%) million short reads (shown in SI method Table 1 and Figure S1A) for the 4 cell lines, and covered about 1436.19, 995.10, 902.70, and 872.01 million bp of sequence data for cell lines 2A1, 2A5, 3A1 and 4A5a, respectively.

RPKM was used as a measure of expression level [3]. The number of Refseq genes with measurable RPKM was 20936, 17266, 17202, 16763 and 16419 for JF1, 2A1, 2A5, 3A1 and 4A5a, with median expression at 2.51, 2.82, 3.45, 3.83 and 3.27 RPKM respectively (SI methods Table 2). The Pearson correlations for expression among the 4 hybrid cell lines were very high, ranging from 0.91 to 0.94 and were highly significant from t-tests at *p*-value < 0.001. The Pearson correlations between the JF1 and the hybrid cell lines ranged from 0.74 to 0.78 and were also significant at *p*-value < 0.001 (Figure S1 B).

**3. Statistical analysis of monoallelic expression**

As stated above, multiple JF1 SNPs were found for a substantial number of Refseq genes. In order to consider allele-specific expression at the gene level, multiple SNPs within a gene were pooled together to obtain the proportion of reads showing the B6 or JF1 sequence. Assuming $K$ SNPs are identified for JF1 and the short reads for a hybrid cell line cover $k(k<=K)$ SNPs, for the $n_{i}$ short reads covering SNP $i(i=1,\cdots,k)$ there are $x_{i}$ nucleotides with the B6 base and $y_{i}$ nucleotides with the JF1 base, where $x_{i}+y_{i}=n_{i}$ and $\sum_{i=1}^{k} n_{i}=n$. We call $n_{i}$ the depth of coverage at the $i$-th SNP and $n$ the pooled depth of coverage across available $k$ SNPs. Define$P_{B6}=\sum_{i=1}^{k} x_{i}/n$, $P_{JF1}=\sum_{i=1}^{k} y_{i}/n$ and let $P_{B6}^{i}=x_{i}/n_{i}$ and $P_{JF1}^{i}=y_{i}/n_{i}$ be the proportion of B6 and JF1 preference at SNP $i$. Statistically, $P_{B6}$ and $P_{JF1}$ are the same as the weighted proportion of the $k$ SNPs in a Refseq gene, with weights$n_{i}$, the depth of coverage for SNP $i$, since

$P_{B6}=\sum_{i=1}^{k} x_{i}/n=\sum_{i=1}^{k} \left(P_{B6}^{i}\times\frac{n_{i}}{n}\right)$ and $P_{JF1}=\sum_{i=1}^{k} x_{i}/n=\sum_{i=1}^{k} \left(P_{JF1}^{i}\times\frac{n_{i}}{n}\right)$.

To avoid sequencing bias where one SNP may have a substantially greater number of reads compared to others, when $n_{i}>365,$ we set $n_{i}^{*}=365$ and kept the B6 and JF1 proportions at SNP$i$ with $x_{i}^{*}=365\times P_{B6}^{i}$ and$y_{i}^{*}=365\times P_{JF1}^{i}$, where 365 is the 80% quantile for SNP depth of coverage combining all cell lines. The exact binomial test was used to test the null hypothesis$H_{0}:P_{B6}=P_{JF1}=0.5$. We defined 4 major classes of refseqs depending on the binomial test and the estimated B6 and JF1 proportions $P_{B6}$and$P_{JF1}$ (see main text).

We defined the quantity$S=(P{}_{B6}-P_{JF1})$, a continuous measure for monoallelic strength and direction, such that genes showing perfect biallelic expression have$S=0$, those with perfect monoallelic expression have $S=1$(B6) or S = -1 (JF1). We clustered Refseq genes and hybrid cell lines by unsupervised hierarchical clustering for matricies of $S$(Refseq genes by cell line), with uncentered correlation and complete linkage. The heat maps were used to demonstrate clustering and the monoallelic strength $S$for genes with monoallelic expression on chrX and autosomes (Figure 2).

**4. Overview of genes with monoallelic expression in hybrid cell lines**

The number of genes satisfying our evaluability criteria (RPKM>=3 and pooled SNP depth >=10) (7133, 7373, 7354 and 6935 genes in cell lines 2A1, 2A5, 3A1 and 4A5a respectively), is shown in SI Methods Table 3. Among these, the numbers of genes with monoallelic expression, a trend towards monoallelic expression, ballelic expression or an uncertain category are summarized in Table S1, together with the FDR for each group [4].

**5. RT-PCR validation of results and concordance between undifferentiated and differentiated cells.**

To evaluate the concordance of RT-PCR results between undifferentiated NSCs, astrocytes and neurons, we used the dichotomous outcome of monoallelic expression (allele preference >=85%) vs. biallelic expression (allele preference <85%), comparing astrocytes and neurons vs. undifferentiated NSCs. Assuming that 10% of genes show random agreement, we calculated the *p*-value as the probability of equal or higher concordance under the null hypothesis that the agreement is caused by random agreement rather than the intrinsic character of the genes. Of the 13 genes assayed, 12 genes (all but *Kcnma1*) showed statistically significant concordance between NSCs and differentiated astrocytes and/or neurons. (Table 1, Table S3).

**References**

[1] Li H. and Durbin R. Fast and accurate short read alignment with Burrows-Wheeler Transform. Bioinformatics, 25:1754-60 (2009)

[2] Li H., Handsaker B., Wysoker A., Fennell T., Ruan J., Homer N., Marth G., Abecasis G., Durbin R. and 1000 Genome Project Data Processing Subgroup The Sequence alignment/map (SAM) format and SAMtools. Bioinformatics, 25, 2078-9. (2009)

[3] Mortazavi A, Williams BA, McCue K, Schaeffer L and Wold B, Mapping and quantifying mammalian transcriptomes by RNA-seq. Natural Methods, 5,621-628 (2008)

[4] Storey JD. A direct approach to false discovery rates. Journal of the Royal Statistical Society, Series B, 64: 479-498. (2002)

Tables

SI Methods Table 1: Number of short reads sequenced and mapped with different criteria.

|  | **Total Sequenced**  **(bp covered)** | **Total # of sequence mapped** | **# sequence uniquely mapped mapQ >=1** | **# of sequence uniquely mapped mapQ >=1 without gap**  **(bp covered)** |
| --- | --- | --- | --- | --- |
| **JF1** | 132,765,082  (5,627,199,560) | 46,421,228 | 34,915,833 | **34,395,475**  **(1,481,323,760)** |
| **2A1** | 62,802,115  (4,978,883,909) | 22,653,521 | 18,255,825 | **17,703,842**  **(1,436,187,609)** |
| **2A5** | 58,900,334  (4,712,026,720) | 15,850,059 | 12,658,090 | **12,438,770**  **(995,101,600)** |
| **3A1** | 40,593,686  (3,247,494,880) | 14,586,339 | 11,601,132 | **11,283,711**  **(902,696,880)** |
| **4A5a** | 36,869,079  (2,894,086,944) | 14,330,313 | 11,506,287 | **11,110,739**  **(872,014,966)** |

SI Methods Table 2: Number of Refseq genes expressed and their quantile distribution for all cell lines.

| **Quantile for refseqs RPKM** | **JF1** | **2A1** | **2A5** | **3A1** | **4A5a** |
| --- | --- | --- | --- | --- | --- |
| **Min** | 0.002 | 0.002 | 0.004 | 0.001 | 0.002 |
| **25%** | 0.244 | 0.210 | 0.306 | 0.330 | 0.263 |
| **50%** | 2.512 | 2.815 | 3.451 | 3.832 | 3.268 |
| **75%** | 14.660 | 18.510 | 18.210 | 19.330 | 18.350 |
| **max** | 17950.000 | 9658.000 | 10790.000 | 8838.000 | 11460.000 |
| **Total # refseq genes sequenced** | 20936 | 17266 | 17202 | 16763 | 16419 |

SI Methods Table 3: Number of Refseq genes satisfying evaluation criteria: RPKM>=3 and pooled SNP depth of coverage >=10.

| **rpkm** | **Hybrid cell lines** | | | |
| --- | --- | --- | --- | --- |
|  | **2A1** | **2A5** | **3A1** | **4A5a** |
| **rpkm>=3** | 8515 (50.00%) | 8869 (52.16%) | 8858 (53.44%) | 8359 (51.56%) |
| **pooled snp depth > =10** | 7133 (41.78%) | 7373(43.18%) | 7354 (43.99%) | 6935 (42.50%) |
| **pooled snp depth < 10** | 130 | 216 | 243 | 234 |
| **No SNP** | 1252 | 1280 | 1261 | 1190 |
| **0<rpkm <3** | 8751 | 8333 | 7905 | 8060 |
| **Total # refseq genes sequenced** | 17266 | 17202 | 16763 | 16419 |
